# Plasmepsins as Antimalarial Drug Targets—Then, Now, and the Future

**DOI:** 10.1002/med.70065

**Published:** 2026-06-02

**Authors:** Brad E. Sleebs

**Affiliations:** ^1^ The Walter and Eliza Hall Institute of Medical Research Parkville Victoria Australia; ^2^ Department of Medical Biology The University of Melbourne Parkville Victoria Australia

**Keywords:** antimalarial, aspartyl protease, malaria, plasmepsin, *Plasmodium*

## Abstract

Malaria is a devastating disease caused by *Plasmodium* parasites. *Plasmodium* parasites express ten cathepsin D‐like aspartyl proteases, called plasmepsins (PMs). These PMs have diverse roles fulfill diverse functions throughout the parasite's lifecycle, though several exhibit functional redundancies. Among them, PMV, PMIV, and PMX are essential for asexual stage development, positioning them as prime candidates for antimalarial development. This review synthesizes current knowledge on PM biology and highlights the pivotal role of PM inhibitors in validating these proteases as therapeutic targets. Learnings from past research on PMs coupled with advances in experimental approaches have largely steered drug discovery efforts toward the development of dual PMIX/PMX inhibitors. Highlighted is an overview of the developmental trajectory of PM inhibitors, their progress to date, and their potential integration into future antimalarial therapies aimed at both prevention and treatment.

**Abbreviations**
ACT: artemisinin combination therapy
AMA1: apical membrane antigen 1
ASP: apical sushi protein
BACE1: beta‐site app‐cleaving enzyme 1
CALM: chemo‐attenuated liver merozoites
CelTOS: cell‐traversal protein for ookinetes and sporozoites
CpASP: *Cryptosporidium* aspartyl protease
CSP: circumsporozoite protein
CyRPA: cysteine‐rich protective antigen
DPAP3: dipeptide amino peptidase 3
DHFR: dihydrofolate reductase
ER: endoplasmic reticulum
EBA175: erythrocyte binding antigen 175
GECO: gametocyte erythrocyte cytosolic protein
GAP: genetically attenuated parasite
GEXP: gametocyte exported protein
HA: hemagglutinin
HAP: histo‐aspartyl protease
HEA: hydroxyethylamine
KAHRP: knob associated histidine rich protein
EMP: erythrocyte membrane protein
IP: imino pyrimidinone
IFP: imino pyrimidinone fused pyrrolidines
LISP2: liver‐specific protein 2
LSA3: liver stage antigen 3
LAI: long‐acting injectable
MEDLE2: mucin‐like enhanced by differentiation lectin‐like extracellular exported 2 protein
MVP1: microvilli protein 1
MIR: minimum inoculum of resistance
MSP1: merozoite surface protein 1
MTRAP: merozoite thrombospondin related anonymous protein
PEXEL: *Plasmodium* export element
PM: plasmepsin
PNEP: *Plasmodium* negative exported protein
PTEX: *P. falciparum* translocon of exported proteins
PV: parasitophorous vacuole
PVM: parasitophorous vacuole membrane
RALP1: rhoptry‐associated leucine zipper‐like protein
RAMA: rhoptry‐associated membrane antigen
RAP1: rhoptry associated protein 1
RBC: red blood cell
ROM4: rhomboid‐like protein 4
RON: rhoptry neck protein
SERA5: serine‐rich antigen 5
SLTRiP: sporozoite‐ and liver stage‐expressed tryptophan‐rich protein
SPAQ: sulfadoxine‐pyrimethamine and amodiaquine
SUB1: subtilisin 1
TCAMs: Tres Cantos antimalarial set
TEXEL: *Toxoplasma* export element
TgASP: *Toxoplasma* aspartyl protease
VTS: vacuolar targeting sequence

## 1 Introduction

Malaria is a life‐threatening disease caused by the infection of protozoan parasites from the genus *Plasmodium*. In 2025, *Plasmodium* parasites caused over two hundred million infections and 597,000 deaths worldwide [1]. The two most lethal forms that cause malaria are *P. falciparum* and *P. vivax*. *P. falciparum* is hyper‐endemic in Africa and causes approximately 90% of deaths. *P. vivax* is responsible for recrudescent infection after activation of dormant liver‐stage hypnozoites that re‐establish the symptomatic blood‐stage of infection many months after the initial infection or treatment [2]. *P. malariae* and *P. ovale* cause mild symptomatic signs of infection; death is rare, and they are not viewed as a public health threat [3]. *P. knowlesi*, a simian zoonotic parasite, is now considered the most common cause of malaria in Malaysia and is becoming increasingly common throughout Southeast Asia [4].

The *P. falciparum* parasite has a complex lifecycle involving a mosquito and a human host. Transmission occurs when an infected female *Anopheles* mosquito takes a blood meal on the human host, releasing saliva that contains anticoagulants and infectious sporozoites. Sporozoites enter the bloodstream and migrate to the liver, where they invade a hepatocyte and replicate to produce schizonts filled with thousands of merozoites. Upon maturation, merosomes containing merozoites bud off from the hepatocyte membrane and enter the systemic circulation. The merozoites are then released and immediately invade an erythrocyte, initiating the asexual blood stage. Over multiple rounds of asexual replication, the parasite proliferates within the erythrocyte, leading to their depletion and vascular sequestration, resulting in the symptomatic manifestations of malaria. Occasionally, during asexual blood stage replication, a subset of parasites differentiates into sexual gametocytes. These forms mature in the bone marrow, where they evade the host immune system and are subsequently transmitted to the *Anopheles* mosquito during a blood meal. Within the mosquito midgut, gametocytes transform into male and female gametes, which fuse to form diploid zygotes. The zygotes develop into motile ookinetes that penetrate the basal lamina beneath the midgut epithelium, forming oocysts. Oocysts undergo meiosis to generate numerous haploid sporozoites, which migrate to the mosquito salivary glands. salivary glands. In the salivary glands, the parasite is primed for transmission to a new human host, perpetuating the cycle of infection.

The multifaceted lifecycle of the malaria parasite presents significant challenges for both fundamental research and the development of effective interventions. Despite these difficulties, therapeutic strategies that target multiple stages of the parasite's development remain critical to achieving disease elimination in endemic regions. There are several interventions aimed at disrupting the parasite's lifecycle. Preventive measures, including bed nets, insecticide spraying, environmental management such as removal of stagnant water, and improved access to antimalarial therapies, have contributed to a marked reduction in the global incidence of malaria since the turn of the century [1]. More recently, two vaccines were approved and deployed across 70 countries [5]. Among these, the R21/Matrix‐M vaccine has demonstrated the highest efficacy, reaching up to 77%. However, clinical data indicate that immunity wanes over time [6]. Despite these advances, malaria continues to pose a major global health burden, with the greatest impact observed in Africa and Southeast Asia.

Antimalarials, such as chloroquine, have been used successfully to treat millions of malaria infections, but the emergence and spread of resistance have necessitated the introduction of artemisinin combination therapies (ACTs) [7]. Partial resistance to ACTs has been reported in Southeast Asia [8] and, more recently, in sub‐Saharan Africa [9]. The partial resistance is primarily characterized by delayed parasite clearance following treatment [10]. Importantly, despite these emerging resistance patterns, ACTs remain effective and continue to serve as the cornerstone of malaria treatment. Nevertheless, the potential for novel resistance mechanisms to arise poses a significant threat to the long‐term efficacy of these frontline therapies. This underscores the urgent need to develop new antimalarial therapies that can safeguard against future resistance and ensure sustained treatment effectiveness. Importantly, new antimalarials should have a novel mechanism of action to mitigate cross‐resistance to existing antimalarial therapies and have a high barrier to the development of resistance [11]. Additionally, new antimalarials should target multiple stages of the parasite's lifecycle to expand their therapeutic application beyond curative application, for inclusion in chemopreventative or transmission‐blocking therapy [12]. Recent efforts to develop antimalarials with these properties have produced several promising candidates that have entered clinical development [13, 14, 15]. Unfortunately, the clinical development of some of these candidates has been halted due to adverse toxicology and limited pharmacokinetics [14, 15], underlining the need to populate the antimalarial clinical portfolio with novel antimalarials.

Sequencing of the *Plasmodium* genome [16] together with *piggyBac* saturation mutagenesis [17] has greatly facilitated the identification and validation of protein targets for their potential as antimalarial drug targets. These approaches have revealed numerous proteases in *Plasmodium* that are essential for parasite development. Proteases are categorized into distinct classes based on their catalytic mechanism or the amino acid responsible for the enzymatic activity, with the major groups including metallo, serine, cystine, and aspartyl proteases. Like many eukaryotes, including mammals, *Plasmodium* expresses multiple members across each of these classes. Recent studies have highlighted the critical and specialized roles of several aspartyl proteases in parasite biology, sparking considerable interest that these proteases represent unique opportunities to develop novel antimalarials.

Common to all aspartyl proteases is the presence of two aspartate amino acids in separate DT/SG motifs. The two aspartates are essential to the catalytic function of the protease. The two aspartates activate a water molecule to act as a nucleophile to attack the carbonyl of the amide peptide bond, resulting in proteolysis. Most aspartyl proteases are endopeptidases and have a preferred specificity for a substrate or peptide sequence, which is determined by the amino acid makeup of substrate‐binding pockets on either side of the catalytic dyad. A structural feature present in all aspartyl proteases is the flap that is defined by a sequence of amino acids, which contains either a phenylalanine or tyrosine acting as a switch to open and close the flap. The flap in the position allows peptide substrates to enter the substrate cleft while simultaneously closing the flap to cover the substrate, allowing proteolysis to occur. The proteolysis products are then released upon opening of the flap.

*P. falciparum* expresses 10 cathepsin D‐like aspartyl proteases known as plasmepsins (PMs), that possess two DT/SG motifs, except for PMIII, named histo aspartyl protease (HAP) that has one DT/SG motif replaced by HTA (Figure 1). The sequence similarity of PMs across *Plasmodium* species is highly conserved, indicating comparable substrate specificity and functional roles among species. Phylogenetic analyses reveal distinct evolutionary clades of PMs: clade A. PMs I–IV; clade B. and E. PMs VI and VIII and PMVII; clade C. PMs IX and X; and clade D. PMV [18]. Notably, PMV exhibits a divergent sequence identity, distinguishing it from the other PM groups. The organization of these clusters reflects their structural architecture, subcellular localization within the parasite, and functional specialization. PMs I–IV are compartmentalized within the digestive vacuole, where they play a central role in hemoglobin digestion [19]. PMIX and PMX are localized to organelles that house enzymes required for merozoite invasion and egress of the RBC [20, 21]. In contrast, PMs VI–VIII are absent during the asexual stage but are thought to be expressed at specific developmental stages within the mosquito vector [22]. PMV is uniquely localized to the ER [23], where it is required for protein trafficking [24, 25]. The functional importance of each PM across different stages of the *Plasmodium* lifecycle represents a central theme of this review.

**Figure 1:**
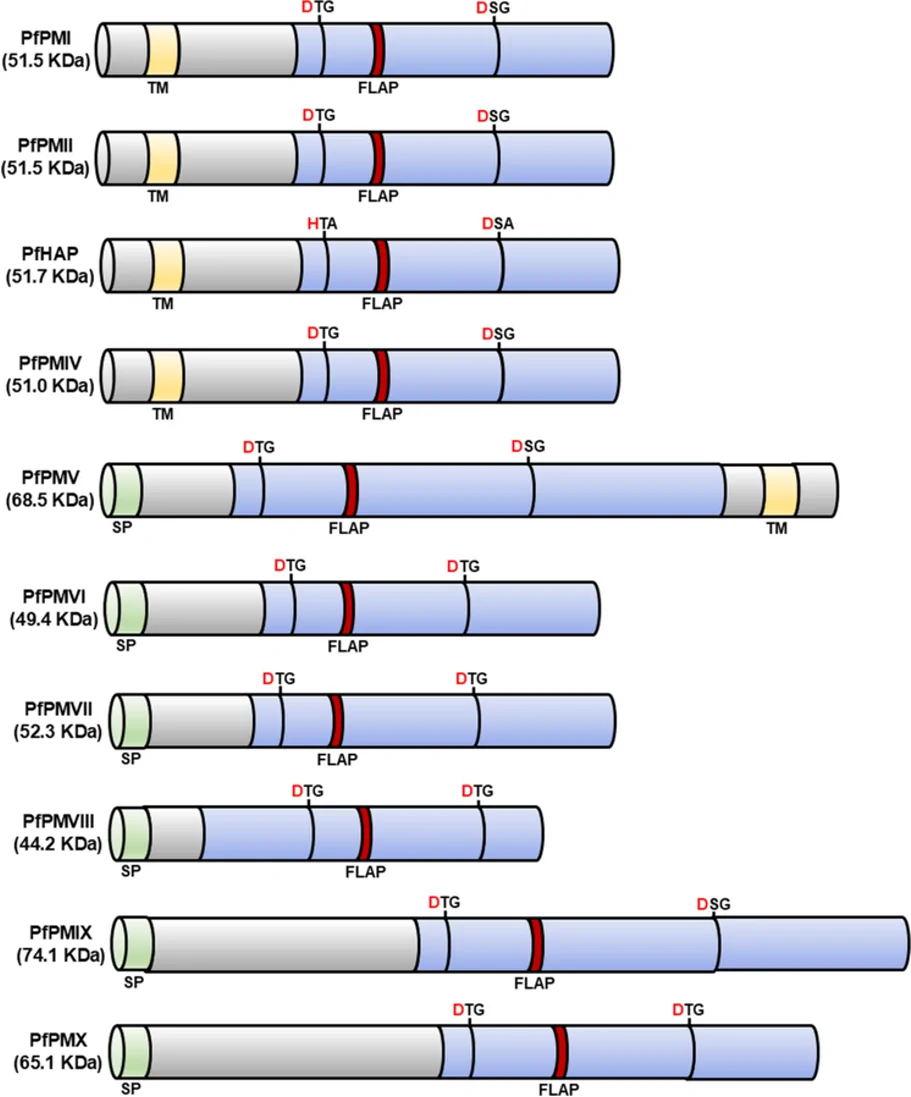
Cartoon representation of the PMs. Putative or known pro‐domains are in gray, signal sequence in green, transmembrane domains in yellow, conserved aspartyl protease domains in blue, conserved catalytic domains denoted by DTG and DSG (and HTA), and flap regions are colored red. [Color figure can be viewed at wileyonlinelibrary.com]

One hallmark of an attractive antimalarial target is the divergence in homology between *Plasmodium* and human proteins, which provides a therapeutic window for selective inhibition. Sequence analysis reveals that beta‐site app‐cleaving enzyme 1 (BACE1) shares the greatest similarity with PMs I–V, pepsin and cathepsin D/E with PMIX and PMX, and renin with PMs VI–VIII. However, overall sequence identity between human orthologues and each PM remains low, supporting the potential for selective targeting of PMs by small molecules.

Importantly, substrate recognition is largely determined by the architecture of the binding cleft, which confers specificity for both natural substrates and small‐molecule inhibitors. Consensus substrate sequences therefore serve as valuable indicators of substrate uniqueness and specificity between human and *Plasmodium* orthologues. The putative substrate consensus sequences for PMs I–IV, V, IX, and X are summarized in Table 2. Subtle differences in the substrate preferences of PM V, IX, and X, relative to human aspartyl proteases and distinct from other PMs, suggest that selective inhibitors can be designed to target these enzymes without cross‐reactivity against human counterparts. PM inhibitor selectivity and parasite efficacy form key discussion points of this review.

This review distinguishes itself from recent reviews, which primarily focus on contemporary insights [26, 27], by tracing the pivotal historical findings that shaped our current understanding of PM biology. It also provides a comprehensive analysis of the present landscape on the development of targeted PM antimalarials, offering an integrated perspective on both foundational and translational progress. This review begins by delineating the distinct roles of individual PMs in the development and survival of the *Plasmodium* parasite throughout its complex lifecycle. The limitations of prior research are critically examined and highlight the current gaps in understanding PM biology. Building on current insights into parasite biology, the review will define which PMs represent biologically and chemically validated and pharmacologically promising targets for antimalarial intervention. Finally, the review will outline the challenges and shortcomings of historical and ongoing efforts to develop small‐molecule PM inhibitors and propose strategic directions for future drug discovery initiatives.

## 2 The Essentiality and Role of Plasmepsins Across the Lifecycle of *Plasmodium* Parasites

### 2.1 The Dispensable Role of the Digestive Vacuole Plasmepsins

The *Plasmodium* parasite possesses a specialized organelle, known as the food or digestive vacuole, which contains a variety of proteases that are primarily responsible for the degradation of hemoglobin into peptides and amino acids to sustain parasite growth. PMs I–IV are among the many proteases housed in the digestive vacuole [28, 29, 30, 31]. PMIII is commonly referred to as HAP [32]. The proforms of PMs I–IV are membrane‐associated proteins that traverse the secretory pathway to the parasite surface before being internalized together with host hemoglobin into the digestive vacuole. Once inside the digestive vacuole, the cysteine proteases, falcipain‐2 and falcipain‐3 [33], process the prodomains of PMs I–IV to their soluble and active forms (structure of active form of PMII is shown in Figure 2A) [36]. PMs I–IV have been shown to co‐localize within the food vacuole of *P. falciparum* [32] and were independently characterized using recombinant protein assays, which confirmed their capacity to process hemoglobin and hemoglobin‐derived peptides (summarized in Tables 1 and 2) [30, 31, 37, 38].

**Figure 2:**
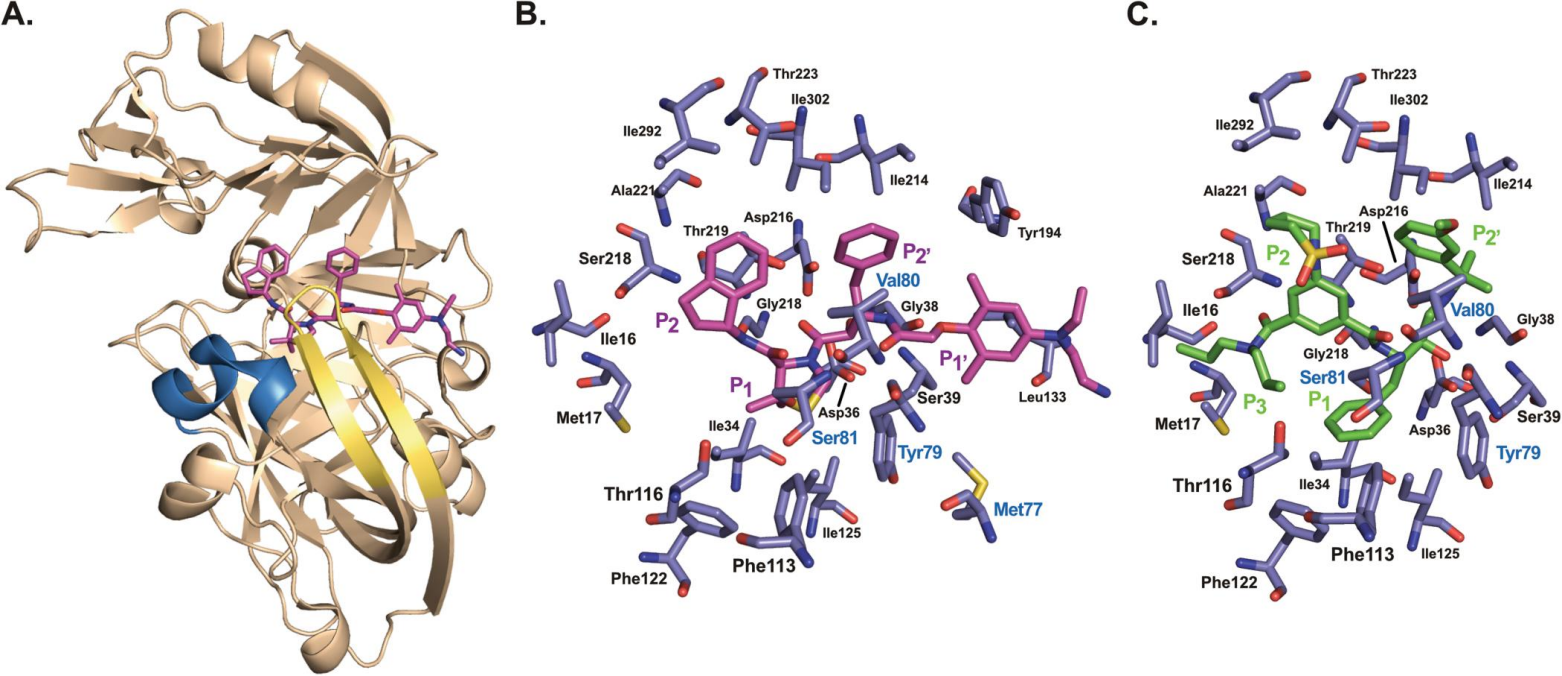
(A) Structure *P. falciparum* PMII in complex with KNI‐10742 (PDB 5YIE) showing the short S_3_ loop (blue) and flap (gold) domains [34]. (B) Close‐up of KNI‐10742 (a close analog of KNI‐1006) with interacting and surrounding amino acids in the catalytic cleft of PMII (PDB 5YIE). (C) Close‐up of TCMDC‐134674 (a close analog of 1*SR*) bound to PMII (PDB 4CKU) [35]. Amino acids numbered as per Reference [34]. Flap amino acids are noted in blue text. [Color figure can be viewed at wileyonlinelibrary.com]

**Table 1:** Role and essentiality of PMs in *Plasmodium*.

PM	Localization in asexual parasites	Role in the asexual stage	Role in the sexual stage	Role in liver stage	Role in the mosquito stage
I–IV	Digestive vacuole	Dispensable in hemoglobin digestion	None	None	Not known. PMIV was reported at the apical tip of the ookinete.
V	ER	Essential for protein export to RBC	Essential for protein export to RBC	Emerging evidence for protein export to the enterocyte	Not expressed
VI^a^	n/a	Not expressed	None	Essential for hepatocyte invasion	Essential for sporozoite development in the oocyst
VII^a^	n/a	Not expressed	Not expressed	Not expressed	Unknown but not essential
VIII^a^	n/a	Not expressed	None	Essential hepatocyte invasion	Essential for egress from oocyst and sporozoite motility
IX	Rhoptries	Essential for RBC invasion	No evidence	No evidence	No evidence
X	Exonemes	Essential for RBC invasion and egress	Essential but precise role is unclear	Essential for merosome or merozoite development	Possible role in mid‐gut invasion.

^a^ Evidence primarily derived from studies on *P. berghei*.

**Table 2:** Drug discovery tools and the validity of each PM as an antimalarial drug target.

PM	Substrates and consensus sequence^b^	Biochemical assay	X‐ray structures	Inhibitors known	Validity as a drug target	Potential therapeutic application
I–IV	Hemoglobin, spectrin. hxF* h	Yes, for all PMs I–IV	PMI: 3QRV, 3QS1. PMII: 1LEE, 1LF2, 1LF3, 1LF4, 1M43, 1ME6, 1PFZ, 1SME, 1W6H, 1W6I, 1XDH, 1XE5, 1XE6, 2BJU, 2IGX, 2IGY, 2R9B, 3F9Q, 4CKU, 4Y6M, 4YA8, 4Z22, 5BWY, 5YIA‐E. HAP: 3FNS, 3FNT, 3FNU, 3QVC, 3QVI. PMIV: 1LS5, 2ANL, 5I70, 5JOD.	Yes, though parasite activity is mediated via PMX or another protein.^a^	PMs I–IV are not valid drug targets	None
V	Numerous. 460 PEXEL proteins predicted. RxL* xQ/E/D	Yes	4ZL4, 6C4G, 8TYF, 8TYG	Yes.^a^ Some also inhibit PMX.	Strong validity. All tools available for drug discovery.	Curative and transmission blocking therapies and likely a causal preventative therapy
VI	Not known	No	None	No	Weak validity. Lack of tools.	Transmission blocking applied in sugar bait
VII	Not known	No	None	No	Not valid.	None
VIII	Not known	No	None	No	Weak validity. Lack of tools.	Transmission blocking applied in sugar bait
IX	RAP1, ASP, RON3, MTRAP, RON6, RALP1, RAP1. SF* hQ/E/D	Yes	None	Yes, though also inhibit PMX.^a^	Clinically validated. All tools are available for drug discovery except an X‐ray structure.	Curative therapy
X	SUB1, AMA1, CelTOS, SUB2, Rh1, Rh2N, Rh2C, Rh4, Rh5, Ripr, PTRAMP, EBA140, EBA175, EBA181. Many more predicted. SF* hQ/E/D	Yes	7RY7, 7TBB, 7TBC, 7TBD, 7TBE, 8DSR, 8TYH, 8W10, 9HB0	Yes^a^	Clinically validated. All tools are available for drug discovery.	Causal preventative, curative, transmission blocking and chemo‐vaccination therapies.

* Denotes cleavage site.

^a^ Refer to Figures 5, 6, 7, 8, 9, 10, 11 for examples.

^b^ x is arbitrary; h is a hydrophobic amino acid.

Early reports positioned the role of PMs within the digestive vacuole as important for parasite development. This view was reinforced by biochemical studies demonstrating inhibition of PMs I–IV with pepstatin and related small molecules, which led the malaria research community to regard these proteases as promising antimalarial drug targets [39]. In retrospect, without the full complement of genetic and phenotyping studies to characterize the essentiality of PMs I–IV, the validity of these proteases as drug targets was questionable. Nonetheless, these initial findings stimulated extensive research efforts, including recombinant expression of PMs and the subsequent design of peptidomimetic inhibitors targeting PMs I–IV. Due to the enormity of the publications describing potent inhibitors PMs I–IV, which have been reviewed extensively elsewhere [40, 41, 42, 43], only a few examples are highlighted in Section 3 for context. At the time, it was perplexing that small molecules that possessed potent broad‐spectrum inhibition of PMs I–IV had little activity against *P. falciparum* asexual stage parasites in vitro. This discrepancy was resolved several years later when techniques to genetically manipulate the parasite emerged [44] and conclusively demonstrated that PMs I–IV are not essential.

Initial knockout studies of the four digestive PMs in *P. falciparum* demonstrated that each enzyme could be deleted with minimal impact on parasite growth [19, 45]. Among them, PMIV appeared most significant, as its deletion resulted in a modest reduction in hemozoin accumulation, suggesting impaired hemoglobin digestion [19, 46]. Subsequent generation of double knockout parasite lines revealed that PM I/IV double mutants remained viable, displaying only a slight growth defect, while PM I/II and PM II/HAP double mutants exhibited normal growth rates. Furthermore, treatment with cysteine protease inhibitors ‐ blocking falcipain‐2 and falcipain‐3 activation of PMs I–IV—showed negligible differences in sensitivity between wild‐type and individual knockout lines. These findings provided the first evidence that all four digestive PMs are non‐essential. A subsequent study extended this research to triple knockouts (PMs I, II, and HAP) and quadruple knockouts of PMs I–IV [47]. The triple knockout parasites were fully viable, while the quadruple knockout displayed only a slight reduction in growth rate and complete loss of hemozoin formation. Collectively, these results indicate that although PMIV contributes to hemoglobin degradation and hemozoin production, PMs I–IV are not essential for asexual parasite survival. Gene deletion studies further highlighted that *P. falciparum* possesses a cohort of digestive vacuole proteases with overlapping functions in hemoglobin catabolism. Since hemoglobin is deficient in several amino acids—including Met, Cys, Gln, and Glu—and entirely lacking Ile, it cannot serve as the sole nutrient source for the parasite. Supporting this, it was demonstrated that in Ile‐only medium, PM I–IV knockout lines exhibited impaired but viable growth [48]. These findings underscore that *P. falciparum* relies not only on hemoglobin digestion but also on alternative nutrient uptake pathways, reinforcing the non‐essential role of PMs I–IV.

Additional evidence for the redundancy of digestive vacuole PMs comes from their distribution across *Plasmodium* species. PMs I, II, and HAP are unique to *P. falciparum* [49], whereas PMIV is the only member conserved in other species. This lineage‐specific expansion is further evidence that PMs I, II, and HAP are dispensable for hemoglobin digestion and parasite survival [50], and highlights the importance of comparative evolutionary analyses when interpreting protein essentiality or target validity. Consistent with this, PMIV knockout parasites in *P. berghei* display a phenotype similar to that observed in *P. falciparum*. In mouse infection models, the *P. berghei* PMIV knockout line was markedly less virulent than wild‐type parasites [51]. The mechanistic basis for this attenuation remains unclear. Intriguingly, mice infected with the PMIV knockout strain developed protective immunity against subsequent challenge with wild‐type ANKA parasites [51, 52]. These findings raise the possibility that attenuated *P. falciparum* PMIV knockout blood‐stage parasites could have potential utility in the development of a chemo‐vaccine, though this prospect requires further investigation.

### 2.2 The Essential Role of Plasmepsin V in Protein Trafficking

PMV performs a key role in protein export to the host erythrocyte in the asexual stage of *Plasmodium*. Transport of hundreds of parasite proteins essential for remodeling are directed to the infected host erythrocyte [53, 54, 55]. Many of the exported proteins have vital roles in the survival of the *Plasmodium* parasite, including evasion of the host immune system and nutrient and waste flux from the host erythrocyte [56]. Approximately, 460 proteins destined for export to the host erythrocyte possess an N‐terminal domain with the consensus sequence RxLxQ/E/D, known as the *Plasmodium* export element (PEXEL) [57], or vacuolar targeting sequence (VTS) [58]. Proteins destined for export are processed by the *Plasmodium* ER resident aspartyl protease, PMV [23], on the C‐terminal side of Leu in the PEXEL (summarized in Tables 1 and 2) [24, 25].

PMV is anchored to the luminal side of the ER membrane via a transmembrane domain [23], and is a part of a secretory complex that includes Sec61 and SPC25 [59]. This complex directs PEXEL‐proteins to exit the ER via a non‐canonical secretory pathway with assistance from an unknown molecular chaperone. The proteins processed by PMV are N‐acetylated before exiting the ER [24], but the precise *N*‐acetylase that performs this task has not yet been identified [60]. The process by which the processed proteins exit the ER is not known, but it is postulated that proteins are likely shuttled to the ER membrane via chaperones, and subsequently, proteins exit the parasite membrane via vesicular transport into the PV [61, 62, 63]. The proteins are then recognized, unfolded, and translocated across the PV membrane into the red blood cell cytosol by a complex of proteins known as the *Plasmodium* translocon of exported proteins (PTEX) [64]. The PTEX is conserved across *Plasmodium* species and is indispensable for asexual parasite development [65, 66, 67]. PEXEL processing by PMV is critical for export of PEXEL‐containing proteins to the host erythrocyte [24, 25, 53, 68, 69]. Indirectly, this processing event is also important for the function of PEXEL‐negative proteins (PNEPs) [54], because PEXEL exported proteins are required for trafficking of PNEPs to the host erythrocyte [53, 56, 70]. There has been much debate on the role of PMV in PEXEL‐dependent export beyond the parasite membrane, which is succinctly covered in a recent review [71].

The PEXEL motif and PMV are highly conserved across *Plasmodium* species [55] and attempts to delete PMV have been unsuccessful, reinforcing its essential role in *Plasmodium* asexual development [24, 25]. The essentiality of PMV in asexual blood stages was recently confirmed using a conditional gene excision [72]. To further demonstrate the importance of PMV in asexual parasite survival and its potential as an antimalarial drug target, peptidomimetics that mimic the PEXEL RxL sequence were generated [73, 74, 75, 76, 77, 78, 79]. These inhibitors will be discussed in more detail in Section 3. Briefly, the peptidomimetics, including WEHI‐916 and WEHI‐842, were used to demonstrate that inhibition of PMV impairs PEXEL processing and protein export, resulting in reduced asexual parasite viability [76, 79], validating PMV as an attractive antimalarial drug target. Furthermore, these PMV inhibitors, that mimicked the PEXEL motif, exhibited high selectivity over human aspartyl proteases, reflecting the uniqueness of the PEXEL substrate cleavage site compared to human aspartyl protease substrate sequences (BACE‐1: SEVxh*; renin: HPF/L*; cathepsin D: hF*Fx). Curiously, using a TetR‐DOZI aptamer system, which permitted tight regulation of PMV, showed that parasites require PMV immediately after RBC invasion, which was not in accordance with protein export via the PTEX [80]. RESA, a protein with a “relaxed” PEXEL, was processed and secreted almost immediately after invasion (0–4 h) [80], providing insight on the possible role of PMV before PTEX components are present for export into the host cell. Further research is needed to deconvolute the role of PMV immediately after invasion, which could further add to the attractiveness of PMV as an antimalarial target.

Protein export also occurs during gametocytogenesis. Both PMV [81] and the PTEX [82] are expressed during the sexual blood stage, confirming that the machinery to execute protein export is present at this stage of development. Proteomic analyses have identified numerous gametocyte‐specific proteins containing PEXEL motifs, and processing of these motifs followed by protein export has been demonstrated [83]. Remarkedly, 10% of proteins upregulated in early gametocytogenesis contain a PEXEL and are predicted to be exported [83]. Fluorescence microscopy has further shown that several PEXEL‐containing proteins are trafficked to the host erythrocyte, including gametocyte exported protein (GEXP) 2, 5, 7, and 10 [83, 84, 85], gametocyte erythrocyte cytosolic protein (GECO) [86], and Pfg744 [87]. GEXPs are thought to function as surface antigens involved in sequestration of gametocytes, while GECO is non‐essential, and the role of Pfg744 remains undefined. Evidence linking PMV activity to protein export and gametocyte development was provided by studies using the peptidomimetic inhibitor WEHI‐842. This tool compound blocked PEXEL processing of GEXP5 in gametocytes and reduced the viability of both early and late‐stage gametocytes [81]. Moreover, WEHI‐842 inhibited parasite transmission to mosquitoes in standard membrane feeding assays, offering the first indication that PMV inhibition disrupts PEXEL processing, gametocyte survival, and transmission [81]. Further investigation is required to establish a definitive connection between PMV function and the export of essential gametocyte proteins with defined biological roles.

Emerging evidence highlights the importance of protein export during the liver stage of *Plasmodium* infection. Studies have demonstrated that the export machinery responsible for erythrocytic protein trafficking is also active during liver stage development. Specifically, PMV motifs in *P. falciparum* liver stages, and components of the PTEX have likewise been detected [88, 89, 90, 91]. Only a limited number of PEXEL‐containing proteins have been shown to undergo export to the host cell, including the sporozoite‐ and liver stage‐expressed tryptophan‐rich protein (SLTRiP) [92], liver‐specific protein 2 (LISP2) [93], circumsporozoite protein (CSP) [94], and liver stage antigen 3 (LSA3) [95]. SLTRiP and LISP2 lack canonical N‐terminal PEXEL motifs, but contain internal PEXEL motifs at positions 264–268 and 373–377, respectively, which may be solvent‐exposed and cleaved to enable export. CSP, in contrast, possesses two RxLxE motifs within its N‐terminal region. Curiously, in *P. berghei*, endogenous LISP2 localizes to the host cell 36 h after hepatocyte invasion [93], whereas in *P. falciparum* LISP2 is restricted to the PV [90]. LSA3 was shown to undergo proteolytic processing and subsequent transport to the PVM. Functional studies further demonstrate that knockdown of either LISP2 or LSA3 impairs liver stage development [91, 95], underscoring the essential role of protein export beyond the parasite membrane. Collectively, these findings suggest that PMV is indispensable for parasite development across its lifecycle within the human host and highlights its potential as an attractive bona fide multistage antimalarial drug target.

Structural insights into PMV have been gained through multiple X‐ray crystallographic studies, which reveal several distinctive features of the enzyme (Figure 3A). PMV possesses a prodomain that is neither proteolytically removed nor conformationally altered by changes in pH, yet its presence does not appear to hinder catalytic activity [23]. PMV, like all cathepsin D‐like aspartyl proteases, has a flap motif with a conserved Phe that functions as a molecular switch regulating substrate binding and release. Uniquely, the flap of PMV also harbors an unpaired Cys [76], the functional significance of which remains unclear. Although positioned awkwardly relative to the substrate‐binding cleft, this cysteine could potentially be exploited in the design of covalent small‐molecule inhibitors.

**Figure 3:**
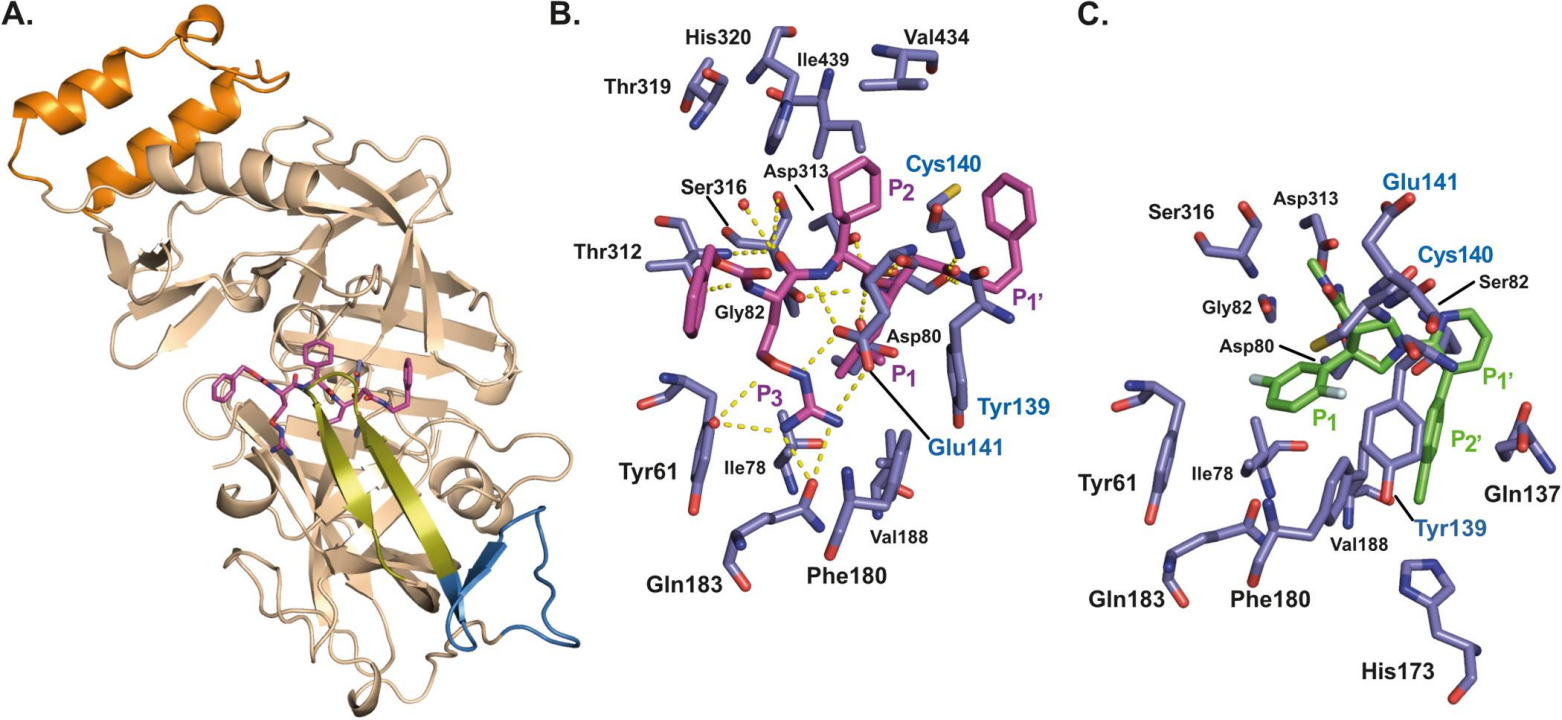
(A) Structure of *P. vivax* PMV bound to peptidomimetic WEHI‐601 (PDB 6C4G) showing the helix–turn–helix domain (orange), nepenthesin domain (blue), flap (gold) domains and catalytic aspartates. (B) Closeup of WEHI‐601 with interacting and surrounding amino acids from *P. vivax* PMV (PDB 6C4G) [77]. *Note:* Thr319 is the position of the T349P mutation found in W601‐resistant *P. falciparum* parasites. (C) Closeup of *S*‐WM48 bound to *P. vivax* PMV (PDB 8TYG) [96]. Flap amino acids are noted in blue text. [Color figure can be viewed at wileyonlinelibrary.com]

PMV further distinguishes itself from other plasmepsins by the presence of a helix–turn–helix motif and a nepenthesin insert (Figure 3A) [76]. Helix–turn–helix domains are typically associated with DNA‐binding proteins [97], and while their role in an ER‐localized protease is uncertain, they may contribute to interactions with trafficking chaperones or translocation complexes. The nepenthesin insert, derived from the plant protease nepenthesin‐1 found in the digestive fluid of *Nepenthesia* pitcher plants [98], contains two disulfide linkages that form a relatively unstructured motif [76]. The nepenthesin insert has been reported to be essential for PMV catalytic activity and parasite development [99], although the precise mechanism by which it regulates enzyme function remains unresolved. Targeting the nepenthesin insert with small‐molecule modulators offers a potential strategy for allosteric inhibition of PMV and the development of novel antimalarial therapies.

### 2.3 The Indispensable Role of Plasmepsin VI in the Mosquito

PMVI is expressed in gametocytes and ookinetes during mosquito‐stage development, but is absent from asexual blood and liver stages [22, 100]. Transcriptional and translational control of PMVI is tightly regulated by repression mechanisms [101] ensuring it is switched on in late‐stage gametocyte development. *P. berghei* parasites with PMVI deleted remained viable through asexual, gametocyte, and gamete stages [100], indicating that PMVI is dispensable for parasite development in the human host. In the mosquito, PMVI knockout parasites transitioned to oocyst formation in the mosquito midgut, although sporulation was impaired, whereby sporozoites failed to develop within oocysts and were absent from salivary glands, indicating PMVI was vital for transmission. Genetic crosses between PMVI knockout and wild‐type gametocytes revealed that PMVI is specifically required for female gametocytes to produce sporozoites, while male gametocyte development proceeds independently of PMVI. Crosses between knockout males and wild‐type females restored sporozoite formation and transmission competence, confirming a female‐specific requirement for PMVI during sporogony. These findings establish PMVI as a critical determinant for *P. berghei* development in the mosquito (summarized in Table 1). Its role in human‐infective *Plasmodium* species, however, remains unresolved. Recapitulating genetic knockouts of PMVI in *P. falciparum* would verify the phenotype and essential role of PMVI. The identity of PMVI substrates and their contribution to sporozoite maturation are currently unknown, though proteomic analyses of sporozoite‐associated factors may help identify candidate substrates. The recombinant expression of PMVI and subsequent biochemical substrate screening could aid in identifying substrates, while target‐based inhibitor development could help probe the role of PMVI in mosquito stage development.

PMVI has emerged as a potential target for transmission‐blocking interventions (summarized in Table 2). One approach involves the use of small‐molecule inhibitors administered to humans, provided these compounds exhibit sufficient metabolic stability and align with the timing of PMVI activity in mosquitoes. Alternatively, PMVI inhibitors could be deployed in sugar baits or impregnated in mosquito bed nets to interrupt transmission in endemic settings [102, 103, 104, 105]. The effectiveness of such interventions, however, would depend on the synchrony between mosquito infection dynamics and bait ingestion.

### 2.4 The Redundant Role of Plasmepsin VII in Gametocytes and the Mosquito Stage

PMVII is not expressed during the asexual blood or hepatocytic stages of the *Plasmodium* lifecycle, but its transcriptional activity in the mosquito stage suggests a potential role in sporogony [49]. Functional analysis using a *P. berghei* PMVII knockout line revealed that the gene is dispensable throughout both asexual and sexual blood stages [106]. Following mosquito feeding on mice infected with the knockout strain, oocyst morphology and midgut oocyst counts were indistinguishable from wild‐type controls. Viable sporozoites were detected in the salivary glands, with no reduction in parasite numbers or transmission efficiency to mice. Furthermore, PMVII‐deficient parasites were able to complete liver‐stage development and initiate blood‐stage infection. Collectively, these findings indicate that PMVII is dispensable for *P. berghei* survival across its entire lifecycle (Table 1). Further investigation is warranted to establish whether PMVII has an indispensable role in *P. falciparum* and other human‐infective *Plasmodium* parasites.

### 2.5 Plasmepsin VIII Is Necessary for Transmission of the Sporozoite From the Mosquito

PMVIII expression is absent during the asexual blood stages and hepatocytic development of *P. falciparum*. PMVIII is co‐localized with granular cytoplasmic structures in stage III to V gametocytes, although it is not detectable in mature gametes [22]. In contrast, *P. berghei* parasites display robust PMVIII expression between days 4 and 8 following mosquito infection, suggesting a potential role in sporogony [107]. Asexual parasites with PMVIII deleted remained viable during asexual development and are transmissible from mice to mosquitoes via blood feeding [107]. Oocyst formation in the mosquito midgut was unaffected, demonstrating that PMVIII is dispensable for midgut colonization and transmission from mice to mosquitoes. However, PMVIII‐deficient sporozoites failed to migrate from the midgut to the salivary glands, despite midgut sporozoite numbers being comparable to wild‐type parasites. This indicates that PMVIII is not required for sporozoite development but is necessary for gliding motility, a process critical for salivary gland invasion and subsequent transmission to the mammalian host (Table 1). The molecular substrates of PMVIII that underpin the defect in motility remain unidentified. The recombinant expression of PMVIII and biochemical substrate screening could help identify PMVIII‐specific processing elements and endogenous proteins associated with sporozoite motility and salivary gland invasion.

If PMVIII proves to be essential for transmission of human‐infective *Plasmodium* species, its utility as a therapeutic target may be limited because effective inhibition would require compounds with sustained bioavailability, potentially lasting more than a week post‐administration in humans, to act during the mosquito stage (Table 2). Alternatively, PMVIII inhibitors could be deployed through sugar baits or incorporated into mosquito bed nets to disrupt sporozoite motility within the mosquito and thereby block transmission in malaria‐endemic regions [102, 103, 104, 105]. Beyond their potential as transmission‐blocking agents, the development of PMVIII inhibitors would also provide valuable tools to identify cellular substrates of PMVIII and clarify its mechanistic role in sporozoite motility and host transition.

### 2.6 Plasmepsin IX Is Required for Red Blood Cell Invasion by *Plasmodium* Parasites

PMIX is conserved across *Plasmodium* species and is expressed during the late stage asexual blood stage [22, 108], although it is absent from gametocyte and mosquito stages [21]. Notably, PMIX is upregulated several hours before merozoite egress from hepatocytes during the liver stage [109]. PMIX colocalizes with secretory proteins RhopH3 and cysteine‐rich protective antigen (CyRPA) in the rhoptry organelle, positioning it to process rhoptry‐associated ligands essential for merozoite invasion (Table 1).

PMIX is essential for parasite development during the asexual blood stage, specifically for merozoite invasion of RBCs, but not for schizont egress [20, 21, 108]. Merozoite invasion is orchestrated through a series of ligand–receptor interactions between parasite and host cell surfaces, during which several ligands are proteolytically processed and shed [110]. Although multiple proteases, or “sheddases,” have been implicated in the cleavage of specific surface proteins [111], the full proteolytic activation cascade underlying invasion remains incompletely defined. Recent studies have demonstrated that PMIX processes several key invasion ligands, including the rhoptry‐associated protein 1 (RAP1), apical sushi protein (ASP), merozoite thrombospondin‐related anonymous protein (MTRAP), rhoptry neck protein 6 (RON6), rhoptry‐associated membrane antigen (RAMA), and rhoptry‐associated leucine zipper‐like protein (RALP1) (Table 2) [108, 112, 113]. Knockdown or small molecule inhibition of PMIX significantly reduces the processing of these ligands, underscoring its critical role in invasion.

PMIX also processes RON3, which is essential in early ring‐stage parasites for protein secretion, localization of the PTEX complex at the parasitophorous vacuole membrane (PVM), and glucose transport [114, 115]. MTRAP is expressed not only in the asexual stage but also in the sexual stage, where it is required for gamete egress [116]. Current data suggests that PMIX is not expressed in gametocytes, therefore it is unlikely to contribute to MTRAP maturation in this context. Whether PMIX plays a role in merozoite exit from hepatocytes during the liver stage, or whether its presence at this stage serves to prime merozoites for subsequent RBC invasion, remains unresolved and will be discussed further in the following section.

PMIX recognizes and processes multiple ligands at sites defined by the putative substrate consensus sequence, SF*hQ/E/D (where h is a hydrophobic amino acid, predominantly Leu or Ile; * denotes the site of cleavage) (Table 2) [113, 117]. These substrates include the autocatalytic activation of its own prodomain, a feature shared with PMX [108, 113]. Bioinformatic searches based on the PMIX consensus sequence may uncover additional substrates; however, PMX exhibits an almost identical consensus motif. Distinguishing between the two proteases, therefore, depends on the subcellular compartments in which their substrates are localized. Such insights could reveal novel proteins involved in the proteolytic cascade that governs parasite invasion. The PMIX substrate sequence has similarities to the human aspartyl protease substrate cleavage sites (BACE‐1: SEVxh*; renin: HPF/L*; cathepsin D: hF*Fx). These sequence parallels could be adventitious in identifying human aspartyl protease inhibitors as starting points for PMIX targeted inhibitor design, but conversely tuning‐out human aspartyl protease activity may be a challenge in finding a suitable selectivity window (see Section 3). Collectively, PMIX plays a critical role in the cleavage of multiple proteins required for merozoite invasion of red blood cells and the establishment of the asexual ring stage, underscoring its importance as a prime antimalarial drug target.

### 2.7 Plasmepsin X Is Required for Parasite Entry Into and Exit From the Host Red Blood Cell

PMX is conserved across all species of *Plasmodium* known to infect humans and is also present in *P. berghei*. PMX is expressed during multiple stages of the *P. falciparum* lifecycle [22], including asexual schizonts, but is absent from ring‐stage parasites and trophozoites. In *P. berghei*, PMX expression has additionally been detected in gametocytes, ookinetes, and hepatocytic schizonts [21]. Within asexual schizonts of *P. falciparum*, PMX co‐localizes with subtilisin 1 (SUB1) in exonemes and with apical membrane antigen 1 (AMA1) and erythrocyte binding antigen 175 (EBA175) in micronemes [20, 118]. Exonemes and micronemes are secretory organelles that release their contents into the parasitophorous vacuole during egress, a process essential for parasite exit from the erythrocyte (Table 1). Further analyses revealed PMX processes microneme and exoneme substrates before discharge, whereas rhoptry substrates are cleaved by PMX after discharge in the PV [118].

PMX plays a pivotal role in both merozoite invasion of erythrocytes and parasite egress from schizonts and infected red blood cells [20, 21, 108]. Asexual stage parasites with reduced levels of PMX exhibit impaired egress and a decreased number of ring‐stage parasites, indicating that PMX contributes to invasion as well. Consistent with these findings, small‐molecule inhibitors of PMX (discussed in Section 3) arrest parasite development at the schizont stage and block RBC invasion.

Egress and invasion are complex processes governed by a proteolytic cascade that activates and matures multiple ligands (Table 2). PMX functions as a central protease within this cascade, processing both proteases and surface ligands important for parasite exit and entry [20, 108, 119]. For example, PMX cleaves the N‐terminal domain of SUB1 [120], thereby activating it to process downstream substrates such as serine‐rich antigen 5 (SERA5) [121] and the merozoite surface protein 1 (MSP1) [122]. The importance of PMX is underscored by studies showing that conditional knockdown or pharmacological inhibition of PMX significantly reduces SUB1 maturation, thereby preventing cleavage of SERA5 and MSP1 [123]. Beyond these substrates, PMX has been shown to process a wide array of proteins critical for invasion and egress [108, 113, 124], which are summarized in Table 2.

PMX processes a wide range of substrates and exhibits specificity for a consensus cleavage sequence that was first defined through proteomic analyses of labeled parasite substrates and later confirmed by biochemical studies [108, 113] and inhibition with peptidomimetics mimicking natural substrates [117]. The putative consensus cleavage sequence for PMX is SF*hE (where h is a hydrophobic amino acid such as Val, Leu or Ile and is commonly Phe; * denotes the site of cleavage) (Table 2). Mining the parasite proteome for this motif in the context of invasion and egress may uncover both known and previously unidentified proteins involved in these processes. Because the PMX consensus sequence closely resembles that of PMIX, substrate assignment will require careful deconvolution based on subcellular localization, whether in exonemes or rhoptries, as well as biochemical validation. Like PMIX, PMX undergoes catalytic autoactivation by processing its prodomain at multiple sites containing SF*hE‐like motifs [113, 125]. The prodomain adopts a distinctive “twisted loop” architecture that occludes the catalytic cleft (Figure 4A), thereby regulating substrate access [125]. In addition to its inhibitory role, the prodomain is critical for correct folding and for trafficking PMX to exonemes and micronemes [118]. In line with PMIX, the substrate consensus sequence of PMX has similarities to human aspartyl protease substrate cleavage sites (BACE‐1: SEVxh*; renin: HPF/L*; cathepsin D: hF*Fx), therefore, the design of PMX inhibitors with high windows of selectivity against human aspartyl proteases has been challenging, although not impossible (see Section 3.5).

**Figure 4:**
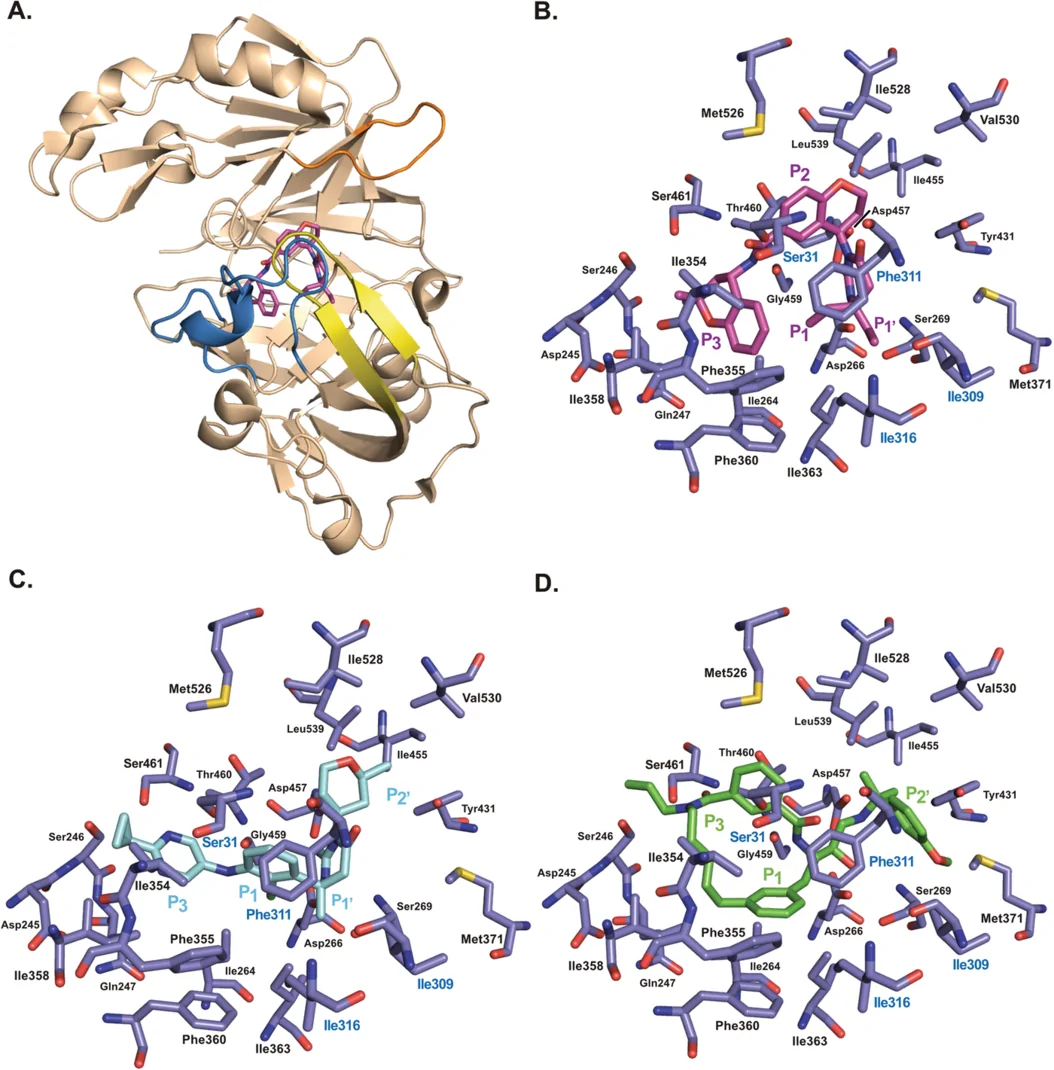
(A) Structure of *P. falciparum* PMX bound to WM382 (PDB 7TBC) showing the S_3_ extended flexible loops (blue), S_2_/S_2_′ extended loop (orange) and flap (gold) domains. (B) Closeup of WM382 and interacting and surrounding amino acids from *P. falciparum* PMX (PDB 7TBC) [113]. (C) Closeup of UCB7362 bound to PMX (PDB 8DSR) [167]. (D) Closeup of macrocycle 7k bound to *P. falciparum* PMX (PDB 9HB0) [169]. Flap amino acids are noted in blue text. [Color figure can be viewed at wileyonlinelibrary.com]

PMX plays a critical role in gametocyte development and transmission to mosquitoes. Inhibitors of PMX block transmission of *P. falciparum* parasites from infected blood and *P. berghei* parasites from infected mice to the mosquito [21, 108, 126]. However, the precise stage at which these inhibitors act remains unclear. Treatment of gametocytes with PMX‐targeting small molecules prevents microgamete formation (exflagellation) but does not affect macrogamete development [21, 126]. The specific substrates cleaved by PMX to enable transmission have not yet been fully defined. One candidate is SUB1, which is processed by PMX in gametocytes [21], while another is the cell‐traversal protein for ookinetes and sporozoites (CelTOS), essential for ookinete traversal of the mosquito midgut epithelium and subsequent oocyst formation [127]. Further studies are required to identify the substrates responsible for transmission and to determine whether PMX inhibitors act primarily at the level of microgamete development, ookinete maturation, or both. Collectively, these findings establish PMX as a bona fide target for the development of transmission‐blocking therapies.

PMX plays an essential role in late hepatocytic stage development [108, 119, 126]. The hallmark of merozoite egress from the liver is the detachment of the infected host cell following rupture of the parasitophorous vacuole, after which merosomes containing merozoites bud from the host plasma membrane and enter the circulation. Treatment with the PMX inhibitor 49c led to a significant reduction in the number of detached hepatocytes in vitro [21]. Notably, 49c did not impair merozoite development but led to the accumulation of parasites within hepatocytes, suggesting that PMX specifically mediates parasite exit rather than maturation. Consistent with this, in mice infected with *P. berghei* sporozoites and treated with PMX inhibitors, liver stage development progressed normally until approximately 50 h post‐infection, but by 55 h, parasitemia was cleared and failed to transition to the asexual blood stage [21, 108, 126]. Similar findings were observed with the PMX inhibitor WM382 in *P. falciparum* humanized mice [109], suggesting that PMX inhibitors were causing a developmental defect in merozoites. It appears that inhibition of PMX, and potentially PMIX, impairs subsequent invasion of red blood cells, thereby preventing establishment of the asexual blood stage. The precise substrates of PMX that govern merozoite maturation and hepatocytic egress remain undefined. SUB1 has been proposed as a likely candidate given its essential role in liver stage exit [128, 129], although further studies are required to identify additional substrates and downstream effectors involved in this process. Collectively, these findings highlight PMX as an attractive chemopreventative target.

The critical role of PMX in merozoite exit from hepatocytes has led to the striking discovery that inhibitors of PMX (and PMIX) may serve as chemo‐vaccination agents. In mice with established *P. berghei* liver stage infections, treatment with dual PMX/PMIX inhibitors (WM382 or MK‐7602) blocked hepatocytic egress and prevented symptomatic malaria. Remarkably, these mice could not be re‐infected with *P. berghei* sporozoites delivered either by injection or mosquito bites, and sporozoites failed to establish liver stage infection for up to 2 years following the initial treatment [109]. This strategy, termed chemo‐attenuated liver merozoites (CALM), highlights the potential of PMX/PMIX inhibition as a durable transmission‐blocking approach [109].

The protective immunity generated by CALM was primarily pre‐erythrocytic, with only partial protection observed against blood‐stage parasites. This may reflect antigen presentation from both liver and blood stages of attenuated hepatocytic merozoites. CSP was identified as the dominant antigen contributing to protection, although additional antigens are likely involved. Whether CALM‐induced immunity in one *Plasmodium* species can protect against blood‐stage infection in another remains uncertain, and cross‐species protection is considered unlikely due to CSP and other antigenic diversity [130]. It also remains unclear whether PMIX inhibition is required for protective immunity, as dual inhibitors were used in these studies. Although PMIX is expressed at the time of hepatocyte exit, it is thought to function primarily in merozoite maturation for RBC invasion rather than hepatocytic egress. Definitive evaluation of this hypothesis will require potent, selective inhibitors targeting PMIX or PMX individually.

An important consideration is how a PMIX/X CALM chemo‐vaccination could be delivered clinically and in the field. Conventional curative treatments are typically administered only after the onset of symptoms, meaning they occur after liver stage infection has concluded, and therefore, CALM chemo‐vaccination may not confer protective immunity. One potential application is the use of PMIX/X CALM as a vaccination agent, analogous to genetically attenuated parasite (GAP) vaccines [131]. However, large‐scale production, formulation stability and field deployment present significant challenges, and this model has yet to be tested experimentally. Mass seasonal administration in endemic regions would be an ideal strategy, though this would require PMIX/X inhibitors with extended half‐lives, similar to the monthly oral dosing regimen of sulfadoxine‐pyrimethamine and amodiaquine (SPAQ) during transmission seasons [132]. The concept of long‐acting injectable (LAI) antimalarials [133, 134], inspired by the success of HIV LAIs that suppress viral titers for up to 6 months [135], has been proposed as a means of delivering seasonal chemoprevention and could also serve as a platform for PMIX/X chemo‐vaccination [109]. An LAI formulation would allow repeated exposures to reinfection during peak transmission periods, effectively providing “booster” immunizations necessary for durable immunity. Moreover, recurrent relapses from latent *P. vivax* hypnozoites during LAI treatment could further reinforce protective responses. The prospect of a PMIX/X CALM strategy, particularly delivered via LAI, represents an exciting advance with the potential to transform malaria control, offering not only protection against symptomatic disease and transmission but also long‐term immunity in endemic populations.

## 3 Inhibition of Plasmepsin Function

### 3.1 Targeting Aspartyl Proteases

Several proteases in *Plasmodium* have been extensively studied and shown to play essential roles in parasite survival. For example, the cysteine proteases falcipains 2 and 3, along with dipeptidyl aminopeptidase 3 (DPAP3), are critical for hemoglobin digestion and erythrocyte egress during the asexual blood stage [136]. Rhomboid‐like protein 4 (ROM4) [137], a metalloprotease, and SUB1 [121], a serine protease, are likewise indispensable for invasion and egress in this stage. Despite their functional importance and distant homology to human orthologues, targeting these proteases with small‐molecule therapeutics remains challenging.

Inhibition of serine and cysteine proteases typically requires a “chemical warhead” capable of trapping the highly reactive nucleophile generated within the catalytic dyad, either reversibly or irreversibly. Similarly, inhibitors of metalloproteases often employ warhead functionalities designed to chelate the catalytic metal ion. While such warheads are highly effective in vitro and serve as powerful probes for interrogating biological systems, their intrinsic reactivity and lack of specificity pose significant liabilities for drug development. Nonetheless, compounds containing these warheads have been successfully developed and approved for use in humans, including Boceprevir and Telaprevir for the treatment of Hepatitis C [138, 139] and nirmatrelvir for the treatment of COVID‐19 [140].

Aspartyl proteases have been historically targeted by inhibitors that possess functionality that also interacts with the catalytic mechanism. Unlike cysteine and serine proteases, inhibition of aspartyl proteases does not require electrophilic “warhead” functionality. Instead, aspartyl proteases are commonly inhibited using transition state mimetics such as statine, bestatin, and hydroxyethylamine (HEA). These chemical motifs contain a hydroxyl group that mimics the transition state of the hydrolytic step in proteolysis and forms hydrogen bonds with the catalytic aspartates, thereby blocking enzymatic activity. Because the inhibitory component does not covalently bind to the catalytic residues, this functionality is referred to as a “head group.” Several PM inhibitors discussed in this review incorporate such transition‐state mimetics.

In designing aspartyl protease inhibitors, transition‐state mimetics are typically incorporated at the P_1_ position of a peptide‐like scaffold. This scaffold provides a framework to mimic the surrounding amino acid residues of the natural substrate, specifically the P_4_‐P_2_ and P_1_′‐P_2_′ positions. While peptidomimetics are valuable tools for probing protease substrate specificity and assessing proteolytic outcomes in cellular models, peptide‐like inhibitors often face limitations, including poor membrane permeability and metabolic instability. To overcome these liabilities, transition‐state mimetics have been integrated into non‐peptide scaffolds. Notably, the HEA motif has been employed in HIV protease inhibitors used in combination antiviral therapies [141], and in renin inhibitors for the treatment of hypertension [142].

HEA‐derived inhibitors proved ineffective in targeting BACE1 for Alzheimer's disease therapy due to their poor penetration of the blood‐brain barrier, a critical requirement for blocking β‐amyloid accumulation in the brain [143]. To overcome this limitation, commitment screening strategies identified several novel head group scaffolds, including cyclic amines and cyclic guanidines [143, 144, 145]. These discoveries led to the development of diverse amino‐pyrimidinone derivatives, which were rapidly adopted by industry and applied to the design of BACE1 and renin‐targeted candidates for the treatment of Alzheimer's disease and hypertension [145, 146]. More recently, cyclic guanidine scaffolds have been repurposed to target the PMs. In this context, achieving selectivity against human aspartyl proteases is essential, as highlighted in recent critical reviews [147, 148]. Of particular importance is the pharmacological inhibition of cathepsin D, due to its role in processing a variety of effectors in the lysosomal compartment.

### 3.2 Inhibitors of the Digestive Vacuole Plasmepsins I–IV

The earliest reported inhibitors of the PMs coincided with the discovery that the PMs I–IV play a central role in hemoglobin degradation within the digestive vacuole, a process that helps sustain parasite development during the asexual blood stage. Originally, each of the PMs I–IV was thought to be functionally redundant [32], and it was therefore assumed that simultaneous inhibition of all four enzymes would be necessary to elicit a phenotypic effect or impair parasite growth. Early peptidomimetic inhibitors were designed based on substrate sequences known to be cleaved by these proteases [149], and although they demonstrated activity, their selectivity and potency varied across PMs I–IV (e.g., KNI‐10006 [150, 151]; Figure 5). Before the genetic knockout studies of the digestive PMs [45], it remained unclear whether inhibition of a single enzyme, or a combination thereof, was sufficient to compromise parasite viability. Furthermore, the level of biochemical potency required to induce parasite death in vitro was not established. Interpretation of these early findings was complicated by the fact that peptidomimetic inhibitors likely exhibited poor membrane permeability and were susceptible to proteolytic degradation within the digestive vacuole, confounding the correlation between PM inhibition and parasite survival.

**Figure 5:**
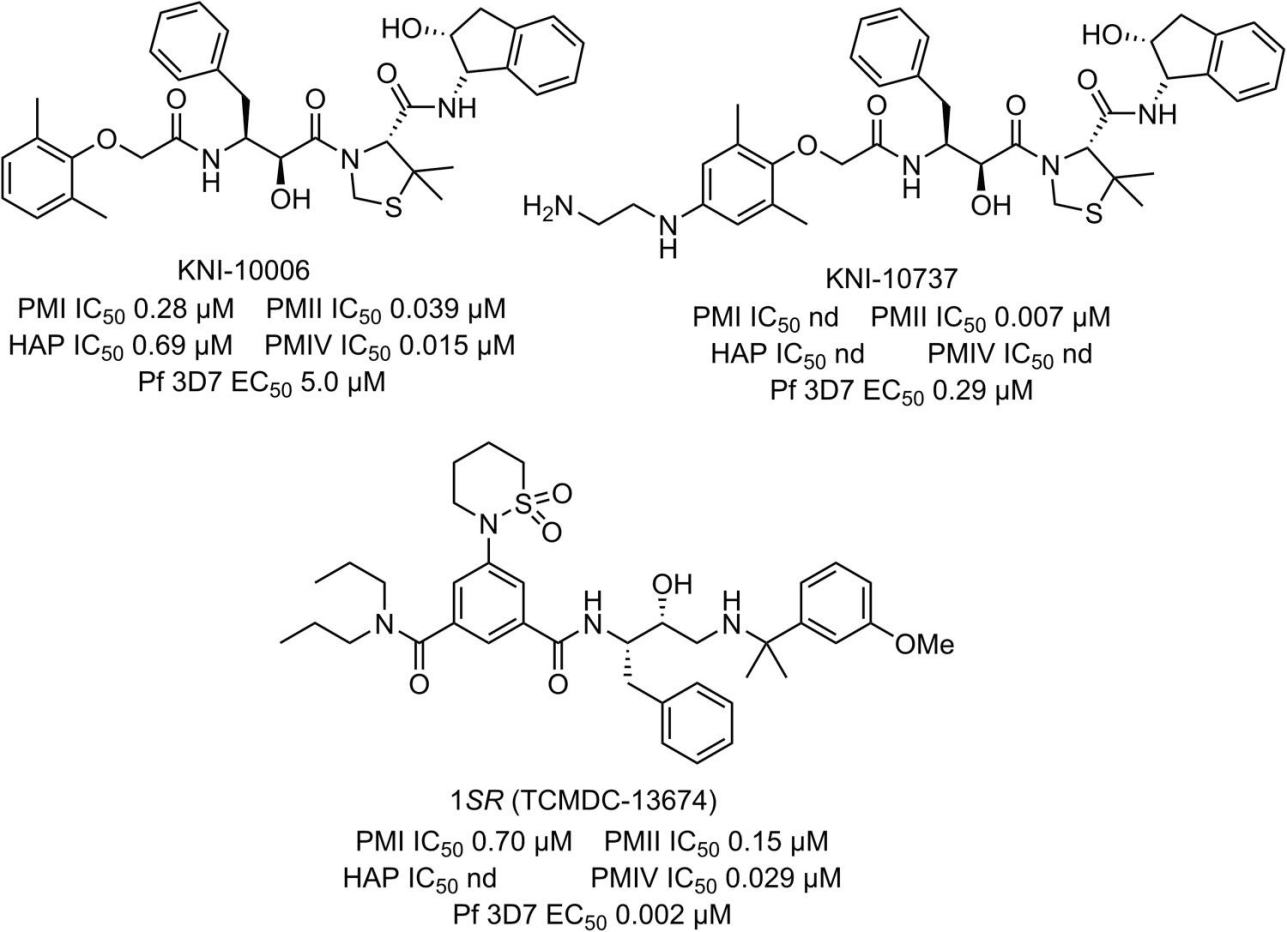
Structures and activity of representative PM I–IV HEA inhibitors.

Several unanswered questions and methodological oversights complicated early efforts to correlate inhibition of digestive PMs with parasite viability. First, many studies reported data for only one or two of the digestive PMs, and parasite viability was assessed for a limited number of compounds. This was likely due to restricted access to recombinant PM proteins and, in some cases, limited facilities for culturing *P. falciparum* to generate growth inhibition data. Second, few studies provided phenotypic or biomarker evidence of on‐target activity within the digestive vacuole. When such evidence was presented, it was typically based on histological preparations of the vacuole, which did not yield quantitative measures of hemoglobin digestion. Moreover, small‐molecule inhibitors were rarely shown to block proteolytic cleavage of hemoglobin or globin, a critical experiment that was performed to establish the role of PMs I–IV in the food vacuole of asexual parasites [32].

Third, studies of PM I–IV inhibitors seldom included appropriate inactive control compounds to confirm that observed effects on parasite viability were directly attributable to PM inhibition. Suitable controls include opposite enantiomers at the α‐hydroxy or α‐carbon positions of peptidomimetic inhibitors, which markedly reduce potency, or analogs with side‐chain substitutions of similar size and polarity that diminish activity. When such compounds were incorporated into structure–activity relationship (SAR) studies, corresponding parasite viability data were often lacking. Additionally, inhibitors and control compounds were not tested in combination with genetic knockdown or knockout parasite lines, which could have provided stronger evidence linking biochemical inhibition to parasite death. Finally, knowledge of other PMs expressed during the asexual stage, such as PMs V, IX, and X, was limited, raising uncertainty as to whether the activity of PM I–IV inhibitors reflected effects on these proteases or unrelated targets. Ultimately, the decisive evidence came when a quadruple knockout of PMs I–IV in a single parasite line was reported [45]. These parasites remained viable, exhibiting only a modest growth defect. Subsequent studies [46, 47, 48, 152] confirmed this finding, demonstrating that inhibition of PMs I–IV alone could not kill the parasite or fully block its development.

Knockout studies of PMs I–IV established their functional redundancy [19, 45, 46], provided the critical evidence that small‐molecule inhibition of the PMs I–IV does not compromise parasite viability. Nevertheless, several inhibitors originally designed to target PMs I–IV were found to exhibit potent activity against *P. falciparum* in culture. This raised the key question: *Which parasite proteins were these compounds acting upon to induce parasite death?* Given that the inhibitors were bona fide aspartyl protease inhibitors, it was hypothesized that they might instead target other essential aspartyl proteases active during the asexual blood stage. While plausible, this hypothesis was difficult to test before the discovery of PMV's role in protein export [24, 25] and more recently the role of PMIX and PMX in invasion and egress [20, 21], In the present day, with the availability of recombinant protein assays, validated biomarkers, forward genetics, knockdown parasite strains, and target engagement technologies, demonstrating on‐target activity has become more feasible and efficient. An alternative hypothesis is that inhibitors of PMs I–IV kill parasites through mechanisms unrelated to aspartyl proteases, particularly in cases where compounds possess non‐traditional head groups. In the following section, several examples of inhibitors initially designed against PMs I–IV are discussed, which likely exert their antimalarial effects via unrelated mechanisms of action.

The earliest inhibitors of PMs I–IV were peptidomimetics designed to mimic the natural substrate sequences of these enzymes, incorporating a transition‐ state mimetic analog at the P_1_ position. Numerous examples of such inhibitors have been reported in the literature and comprehensively reviewed elsewhere [40, 41]. One representative and the more advanced compound from this work was KNI‐10006 (Figure 5) [150]. KNI‐10006 and its analogs are known as adaptive inhibitors that inhibit the primary target, PMII, with the highest potency and maintain similar potencies against PMs I, IV, and HAP [151, 153]. A key change to improve the parasite activity of KNI‐10006 (EC_50_ 5 µM) was the incorporation of an ethylenediamine group, exemplified in compound KNI‐10737 (Figure 5) [153]. Although KNI‐10737 was approximately ten‐fold less potent against PMII compared to KNI‐10006, its activity against parasites improved 17‐fold (EC_50_ 290 nM). X‐ray structural data on KNI‐10742 (a close analog of KNI‐1006) in complex with PMII revealed that the thiazoline group was nestled in the S_1_ pocket, the indane moiety situated in the S_2_ pocket, while the benzyl group occupied the S_2_′ pocket, the phenoxy group protruded out of the S_1_′ pocket in solvent space (Figure 2B). The inclusion of the basic ethylenediamine group on the P_1_′ phenoxy group could potentially affect the physicochemical properties of the compound and, in turn, membrane permeability properties, thereby enhancing parasite activity. Several other modifications within this compound series revealed similar discrepancies between biochemical inhibition of PMs I–IV and parasite activity, suggesting that these inhibitors may exert their antimalarial effects through mechanisms independent of PM I–IV inhibition.

TCMDC‐134674 (1*SR*) emerged as a representative hit from a compound series identified from a high‐throughput screen of the GSK library against *P. falciparum* [154]. TCMDC‐134674 displayed potent activity against *P. falciparum* parasites in a 96‐hour assay (EC_50_ 2 nM) (Figure 5) [35]. The scaffold was noted to contain the classical HEA motif characteristic of aspartyl protease inhibitors, suggesting activity against one or more PMs. Initial speculation proposed that this class could inhibit PMs I–IV, though it was acknowledged that these enzymes might not represent the sole targets. Structure–activity relationship (SAR) studies based on biochemical inhibition of PM I, II, and IV confirmed that the scaffold possessed measurable activity against these proteases. X‐ray structural data enlightened that the dimethyl benzylic motif occupied the S_2_′ pocket, the benzyl group mimicked the P_1_ Phe in the natural substrate located in the P_1_ pocket, while the sultam was accommodated in the P_2_ pocket and the dipropyl carboxamide was situated in the P_3_ pocket of PMII (Figure 2C). The sultam moiety also appeared to perturb flap closing, making the binding cavity more open than compared with other peptidomimetic inhibitors. Consistent with the activity of the 1*SR* compound, the control 1*SS* enantiomer of TCMDC‐134674 exhibited markedly reduced potency, greatly reduced biochemical potency (PM I and II IC_50_ > 10 µM and PMIV, which correlated with diminished parasite activity. Noticeably, the PM I, II, and IV inhibitory activities of this compound class were all significantly less than the reported parasite potency, raising doubts that vacuolar PM inhibition accounted for the observed antimalarial effect. More recently, complementary biochemical, phenotypic, and genetic analyses determined that TCMDC‐134675, an analog of TCMDC‐134674 (1*SR*), killed asexual stage parasites by blocking erythrocyte egress through inhibition of PMX [20]. Thus, providing definitive evidence that PMX is the genuine target of this HEA inhibitor class.

Several inhibitors of PMs I–IV that are not derived from a peptidomimetic‐like scaffold have been reported and reviewed in the literature [42]. One example is the piperidine class, which was originally designed to target the digestive plasmepsins [155, 156]. This compound class binds in the open flap confirmation to PMII in contrast to the closed flap confirmation of peptidomimetic‐derived compounds [157]. Compound 18 and 30 from this series possess potent and modest anti‐plasmodial activity (EC_50_ 0.13 and 0.5 µM) (Figure 6), but the biochemical activity against the PMs potency (PM I, II and IV IC_50_ 1.1, 0.11, and 19.4; and 0.034, 0.019 and 0.007 µM) does not corroborate the cellular potency, so it is plausible these molecules act via a different mechanism of action other than targeting PMs I–IV. The author of this review has demonstrated that this class of molecule does not inhibit PMV (unpublished data) nor demonstrate a 48 h death phenotype correlating to inhibition of PMIX or PMX [20, 21]. Therefore, these compounds likely kill the parasite via a non‐aspartyl protease target.

**Figure 6:**
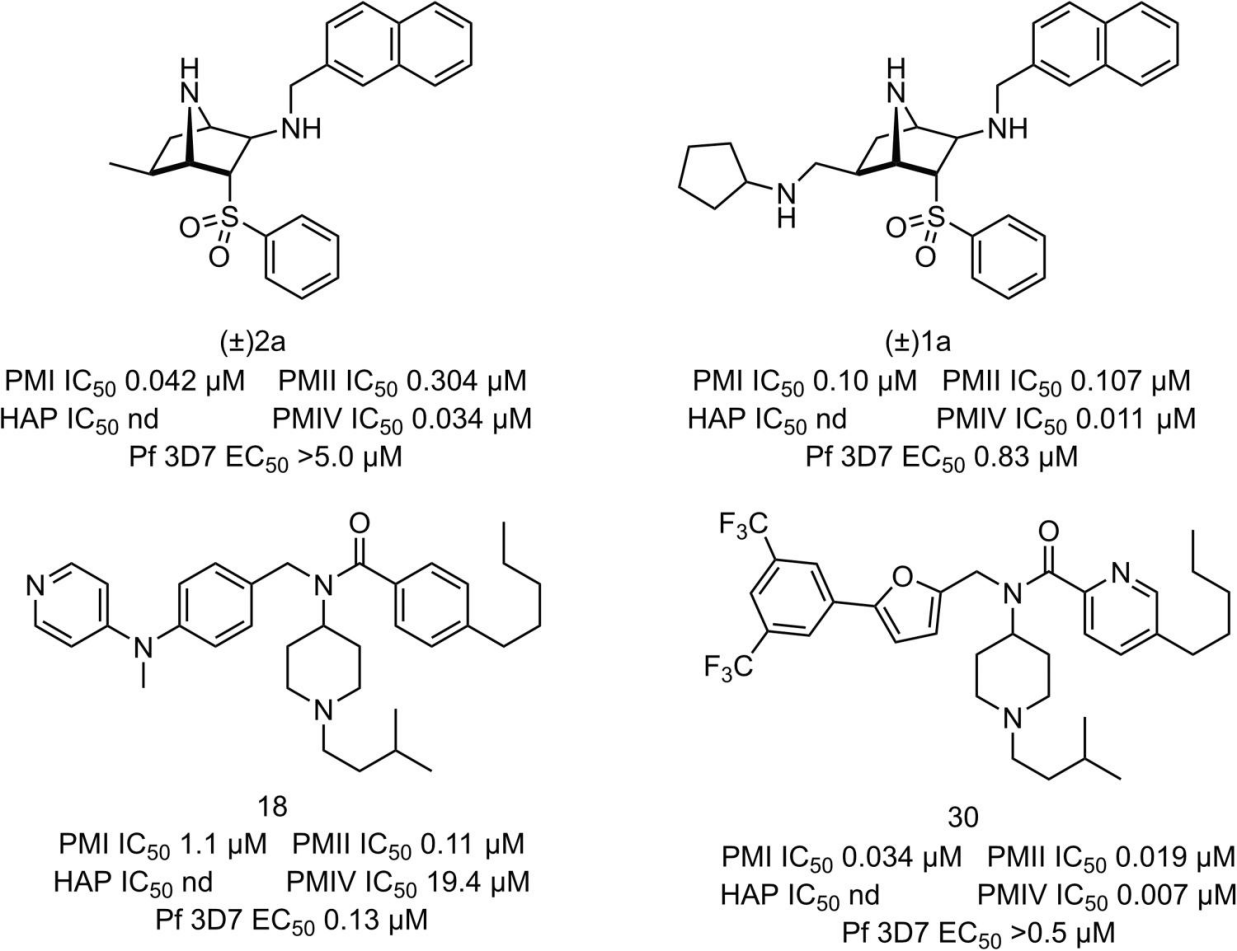
Structures and activity of representative PM I–IV cyclic amine inhibitors.

Another example of a compound class lacking the classic aspartyl protease inhibitor chemotype is the azanorbornene series (Figure 6) [158]. Despite compounds 2a and 1a possessing similar physicochemical properties, they exhibited disparate activities between biochemical PM and parasite assays. These findings would suggest that PMs I, II, and IV are unlikely to be the cellular targets of this compound class ‐ even independent of the knowledge that PMs I–IV are dispensable for parasite survival. Given the limited phenotypic data available, it remains challenging to propose a definitive mechanism of action for this compound series.

The amino hydantoin class has been extensively utilized in drug discovery programs targeting BACE1 in Alzheimer's disease and renin in hypertension. This chemotype was later identified from a high‐throughput screen of the GSK TCAMs library against the *P. falciparum* asexual blood stage parasite [154]. Representative compounds from this class, 8p (CWHM‐117) and TCMDC‐136879, demonstrated potent inhibition of PM II and IV (IC_50_ 0.004–0.045 µM) (Figure 7) [159]. However, the parasite activities differed markedly (EC_50_ 0.46 and 2.76 µM), raising questions on whether the observed antimalarial effects were truly attributable to inhibition of PM II and IV. TCMDC‐136879 was subsequently tested against PMV and exhibited modest activity (IC_50_ of 977 nM). More recently, it was revealed that 8p (CWHM‐117) inhibits PMX in a biochemical assay (IC_50_ 0.600 µM) (Figure 7) and inhibits parasite egress from the RBC [20], thereby establishing PMX as the genuine target of the amino hydantoin class.

**Figure 7:**
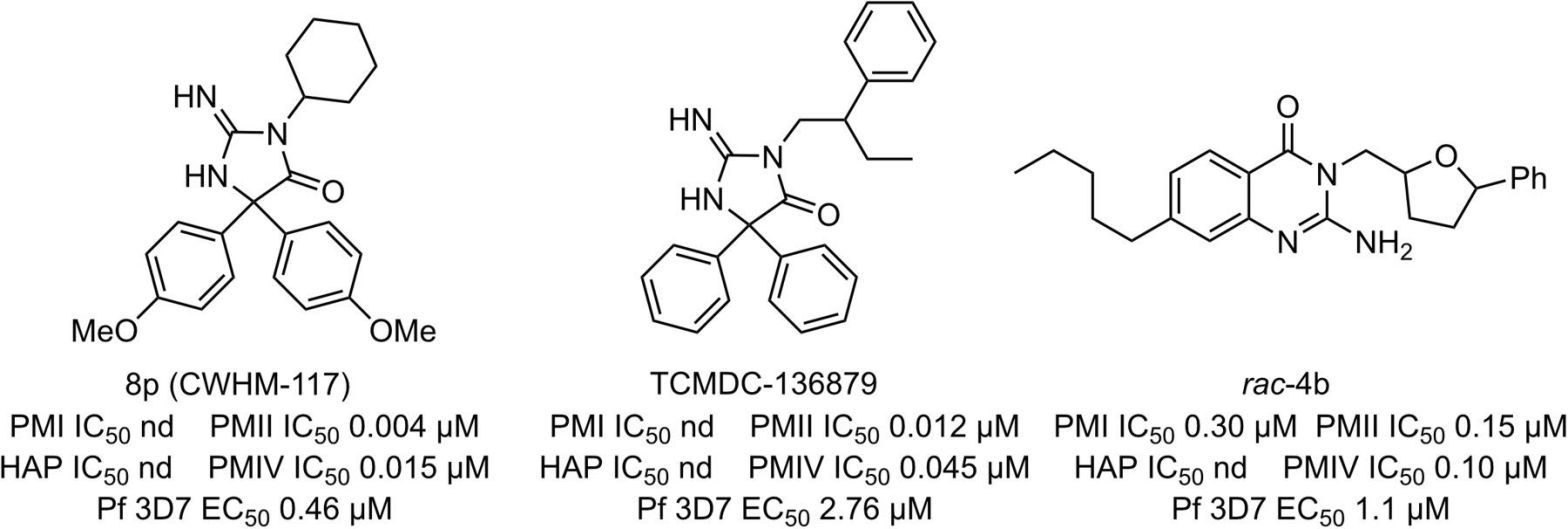
Structures and activity of representative PM I–IV cyclic guanidine inhibitors.

An NMR‐based screen of an Astex fragment library uncovered the 2‐amino quinazolin‐4‐one class with modest inhibition of PMII (IC_50_ 24 µM). Modification of the S_1_′ furfuyl group and S_1_ aryl group resulted in *rac*−4b with potent inhibition of PM I, II, and IV (IC_50_ 0.30, 0.15, and 0.10 µM) and 16‐fold selectivity against human cathepsin D (Figure 7) [160, 161]. *rac*−4b exhibited modest anti‐plasmodial activity (EC_50_ 1.1 µM) but there was uncertainty as to whether this activity was derived from inhibition of the digestive vacuole PMs or another molecular target—likely PMX.

The evidence surrounding PM I–IV inhibitors underscore critical considerations for both target validation and confirmation of on‐target activity within the parasite. As in any chemical biology or drug discovery program, it is essential to employ a comprehensive toolkit to ensure that biochemical inhibition translates into the intended cellular phenotype. The development of PM I–IV inhibitors provides valuable lessons in this regard. Importantly, several historically developed compounds with strong antiparasitic activity may still serve as promising starting points for targeting plasmepsins essential to parasite survival. However, before advancing such candidates, rigorous evaluation using the full spectrum of validation approaches will be necessary to establish definitive on‐target activity in parasites.

### 3.3 Inhibitors of Plasmepsin V That Block Export of PEXEL Proteins

PMV processes the PEXEL motif of hundreds of proteins that are required for their export into the RBC, a pathway essential for host cell remodeling and parasite survival [24, 25]. As such, PMV is viewed as an attractive antimalarial drug target. PMV has distinctive substrate specificity for the RxLxQ/E/D motif, and its distant homology to human proteases suggests that selective inhibition with small molecules should be achievable without significant off‐target effects on human aspartyl proteases. Moreover, this sequence specificity is unique among *Plasmodium* proteases, providing an opportunity to design selective chemical probes that can explore the biology surrounding PMV and consolidate it as a therapeutic target (Table 2).

PMV presents several challenges as a drug discovery target. Firstly, PMV is localized to the ER of the asexual‐stage parasite [23], and is where it is encompassed by four membranes: the erythrocyte, parasitophorous vacuole, parasite, and ER membranes, requiring inhibitors to traverse multiple membranes to target PMV. Secondly, although PMV's unique substrate specificity is attractive from a selectivity perspective, it was unclear whether the P_3_ arginine motif, seemingly necessary for PMV engagement and inhibition, could be effectively mimicked or substituted by alternative functionality [53, 79]. The guanidine group of the arginine side chain is generally avoided in drug discovery due to poor membrane permeability and limited gastrointestinal absorption, which compromise cellular potency and oral efficacy. Finally, PMV is expressed at very high levels during asexual development, and parasites remain viable even with >95% knockdown of PMV [80], indicating that high compound concentrations, far in excess of the biochemical IC_50_ value, would be required for complete inhibition of PMV in parasites.

The first small‐molecule inhibitors of PMV were peptidomimetics designed to mimic the native PEXEL motif (RxLxQ/E/D). These compounds incorporated the transition state mimetic statine and were specifically engineered to reproduce the RxL element of the PEXEL sequence [73, 75, 78]. Subsequently, it was revealed that the xQ/E/D motif could be substituted with hydrophobic groups without loss of potency, highlighting its redundancy in PMV inhibition. The amino acid at the P_2_ position, while not essential for activity, was found to modulate potency and guided the development of WEHI‐916, which mimicked the RVL motif. WEHI‐916 exhibited potent biochemical inhibition of PMV (IC_50_ 0.09 µM) but modest anti‐plasmodial activity (EC_50_ 6.1 µM), a limitation initially attributed to low membrane permeability arising from its peptide‐like characteristics (Figure 8) [79].

**Figure 8:**
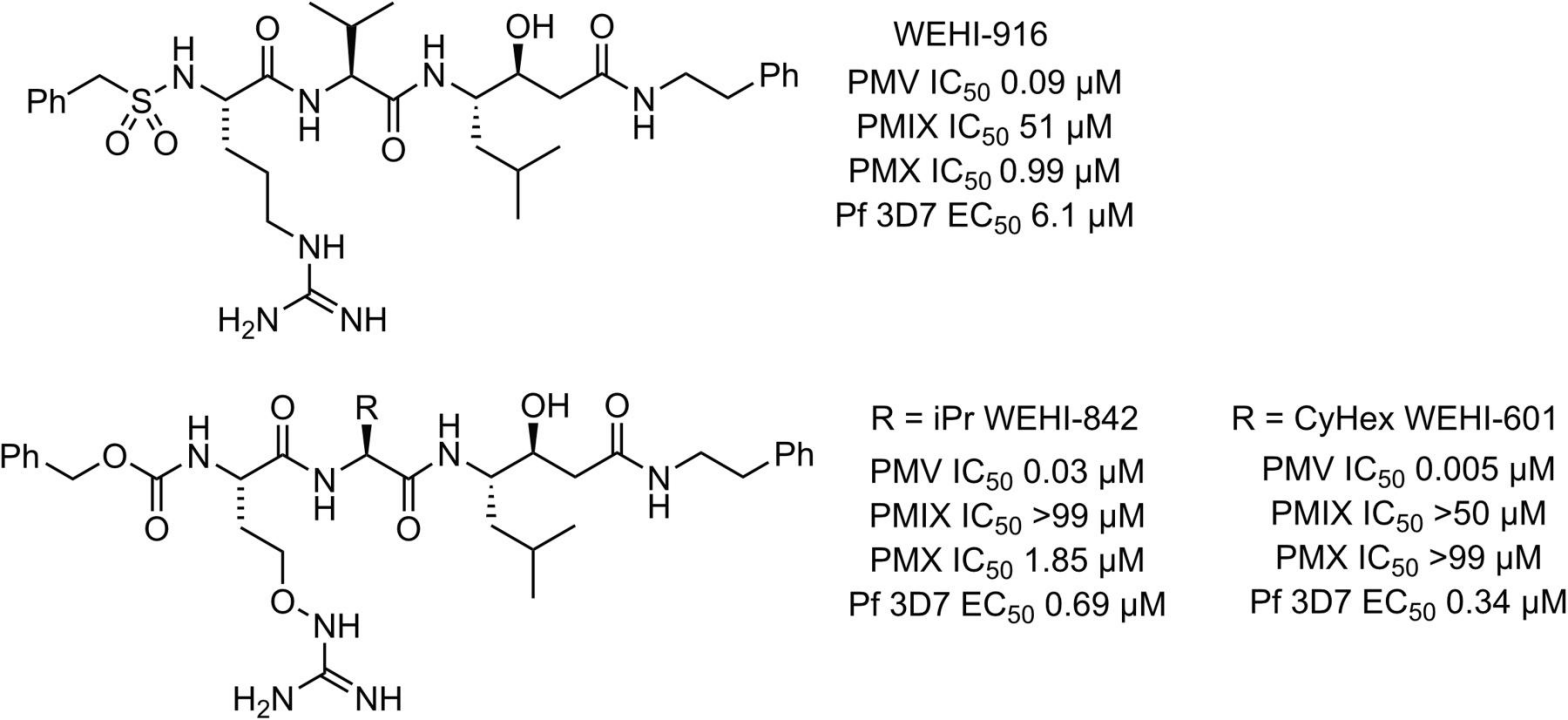
Structures and activity of PMV selective inhibitors.

WEHI‐916 served as a tool compound to characterize phenotypes associated with inhibition of PMV in *P. falciparum* parasites [79]. Treatment with WEHI‐916 resulted in the accumulation of unprocessed PEXEL proteins, including PfEMP3 and KAHRP, and reduced export of the PEXEL protein HYP8 to the RBC. Although PfEMP1 itself is not a PEXEL protein, its presentation on the erythrocyte surface depends on several PEXEL proteins (reviewed in [162, 163); accordingly, WEHI‐916 impaired both its export and surface presentation, as evidenced by reduced erythrocyte adhesion to chondroitin sulfate A. In combination with *glms*‐inducible knockdown, WEHI‐916 showed increased sensitivity relative to the wild‐type 3D7 line, and reduced sensitivity in parasites overexpressing PMV, further supporting its on‐target activity. Negative control compounds WEHI‐024 and WEHI‐025, which exhibited negligible biochemical inhibition of PMV, failed to induce these export phenotypes and displayed markedly reduced antiparasitic activity, underscoring the importance of appropriate controls. Collectively, these findings demonstrated that parasite viability was compromised through inhibition of PMV. Moreover, WEHI‐916 displayed only weak inhibition of human cathepsin D and BACE1 and was non‐toxic to human cells, reinforcing the view that PMV represents a druggable antimalarial target.

Second‐generation peptidomimetic inhibitors of PMV were developed to enhance both biochemical potency and antiparasitic activity. In one key modification, the P_3_ Arg residue of WEHI‐916 was replaced with the bioisostere canavanine (Cav), yielding WEHI‐842. This substitution produced a threefold increase in PMV inhibition (IC_50_ 0.03 µM) (Figure 8) [74, 76]. The reduced basicity of Cav relative to Arg likely improved membrane permeability, translating into a corresponding 10‐fold enhancement in anti‐parasitic activity (EC_50_ 0.69 µM). Structural analysis of WEHI‐842 bound to PMV by X‐ray crystallography [76] provided the first detailed insights into the molecular basis of inhibition and specificity. The X‐ray structure revealed that WEHI‐842 is bound to PMV in a closed‐flap confirmation. The P_3_ Arg formed an intricate network of hydrogen bonds with residues in the S_3_ pocket. The P_1_ Leu fully occupied the S_1_ pocket enclosed with hydrophobic amino acids, while N‐and C‐termini protruded out from the catalytic cleft of PMV (Figure 3B). Guided by these structural data, subsequent optimization focused on the P_2_ position of the peptidomimetics [77]. Incorporation of cyclohexylglycine at this site proved most ideal in fully occupying the S_2_ cavity of PMV (Figure 3B), resulting in WEHI‐601, further enhancing PMV inhibition (EC_50_ 0.005 µM), while also improving blockade of protein export and anti‐plasmodial activity (EC_50_ 0.34 µM) (Figure 8).

Peptidomimetic inhibitors demonstrated a clear correlation between PMV inhibition, blockade of PEXEL processing, and antiparasitic activity, supporting the conclusion that parasite killing occurs via PMV inhibition. However, a marked disparity between biochemical potency against PMV and anti‐parasitic activity (Figure 8) raised uncertainty about the extent of the on‐target activity. To address this, forward genetics and proteomic analyses were performed using WEHI‐601. Resistant parasite lines were selected, and whole‐genome sequencing identified a T371P mutation within the S_2_ pocket of PMV (Figure 3B) [164]. Interestingly, analogs incorporating P_2_ motifs that mimic amino acid side chains such as Val, Leu, and Ile remained active against PMV^T371P^ parasites, indicating that this mutation does not impair processing of PEXEL substrates containing these residues. Consistent with this, PMV^T371P^ parasites exhibited no fitness cost. Further validation using biased and global proteome solvent stabilization confirmed direct engagement of PMV by WEHI‐601 in parasites [164], reinforcing that the antimalarial activity of peptidomimetics results from PMV inhibition. The observed discrepancy between biochemical inhibition and cellular potency is thought to arise from limited membrane permeability of peptidomimetics, but more critically from the elevated expression levels of PMV, necessitating inhibitor concentrations far exceeding the biochemical IC_50_ to achieve parasite killing [80].

The next stage in the development of PMV inhibitors lies in designing compounds with improved drug‐like properties capable of achieving efficacy in mouse models of malaria. Within drug discovery, peptidomimetic scaffolds are generally considered unsuitable for this purpose, and attention has shifted toward identifying drug‐like starting points, following the trajectory established by BACE1 inhibitors that progressed to clinical development. One such scaffold, the amino hydantoin derivative TCMDC‐136879 (Figure 9), was recently reported to exhibit modest PMV activity (IC_50_ 0.977 µM) [159]. Although this compound also showed modest inhibition of PMX, it represents a potential starting point for medicinal chemistry campaigns aimed at developing orally efficacious PMV inhibitors.

**Figure 9:**
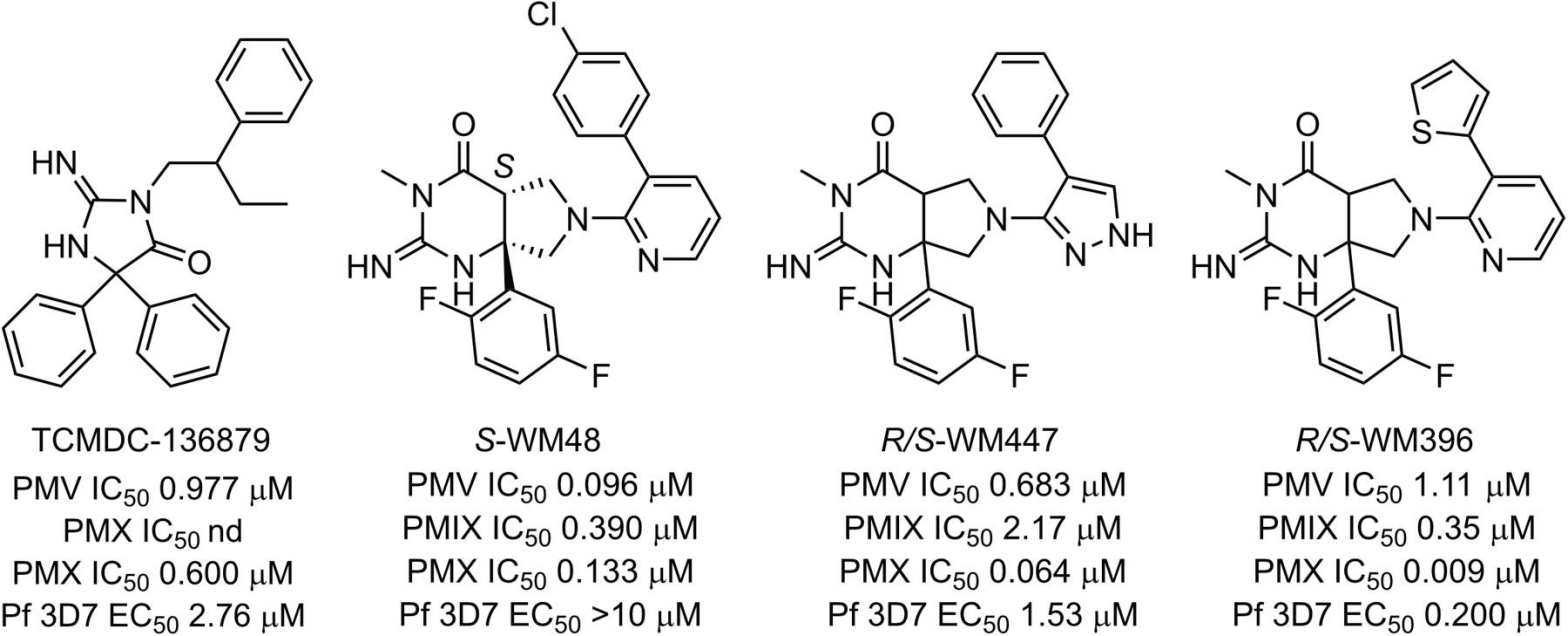
Structures and activity of dual PMV and PMX inhibitors.

More recently, a series of imino‐pyrimidinone‐fused pyrrolidines (IFP) was described as dual inhibitors of PMV and PMX [96]. This scaffold originated from a program targeting human BACE1 for the treatment of Alzheimer's disease [165, 166]. Substitutions on the *N*‐biaryl moiety of the IPF scaffold modulate selectivity between PMV and PMX. For example, *S*‐WM48 potently inhibited PMV (IC_50_ 0.096 µM) and modestly inhibited PMX (IC_50_ 0.133 µM), whereas *R/S*‐WM447 and *R/S*‐WM396 potently inhibited PMX (IC_50_ 0.064 and 0.009 µM) but exhibited reduced potency for PMV (IC_50_ 0.683 and 1.11 µM) (Figure 9). All of these compounds also demonstrated modest inhibition of PMIX (IC_50_ 0.35–2.17 µM). Notably, *S*‐WM48 lacked antiparasitic activity, while *R/S*‐WM477 and *R/S*‐WM396 exhibited modest and potent anti‐plasmodial activity (EC_50_ 1.53 and 0.200 µM), suggesting the anti‐parasitic activity was primarily mediated through PMX inhibition. X‐ray structures of *S*‐WM48 bound to PMV uncovered an open‐flap confirmation where the biaryl motif of *S‐*WM48 extended from the S_1_′ pocket deep into the S_2_′ pocket, while 2,5‐difluroraryl group occupied the S_1_ pocket (Figure 3C). This structural information revealed motifs amenable to modification, particularly engaging with the S_3_ pocket, offering opportunities to design selective PMV or PMX inhibitors, or dual inhibitors targeting both enzymes. As AI and structural modeling software become more advanced and robust, these technologies could assist in the design of a dual inhibitor. A dual PMV/PMX inhibitor would be particularly attractive, as it could provide a high barrier to the emergence of resistance. Nevertheless, the design of PMV inhibitors that combine robust potency with favorable drug‐like properties remains a significant challenge.

### 3.4 Inhibitors of Plasmepsins IX and X Block Parasite Invasion and Egress

PMIX and PMX are essential for parasite development during the asexual blood stage, with PMX also playing critical roles in sexual‐stage maturation and liver‐stage egress [20, 21, 108, 126]. Both proteases are fully validated and considered druggable antimalarial targets [108, 126, 167]. Their close structural homology and shared substrate consensus sequences make dual inhibition feasible, although achieving selectivity for either PMIX or PMX individually may be more challenging. Small‐molecule inhibitors of these enzymes have been instrumental in elucidating their biological functions and establishing their importance in parasite survival.

Tool compounds used to investigate PMIX and PMX were largely identified through screening campaigns of compound libraries against *P. falciparum*. Elucidating their mechanisms of action with parasite phenotypic assays, biochemical studies, and complementary biological tools was critical in confirming that these compounds selectively targeted PMIX and/or PMX, rather than PMs I–IV, as discussed in Section 3.2. Examples include HEA‐derived compounds, such as 49c [156] and TCMDC‐134675 [35, 154], and the amino hydantoins, TCMDC‐136879 and CWHM‐117 (8p) [159]. These compounds exhibited modest to potent anti‐plasmodial activity (Figures 6 and 10). These compounds were found to primarily exert their parasite activity by targeting PMX and were subsequently employed as probes to study PMX and PMIX function.

**Figure 10:**
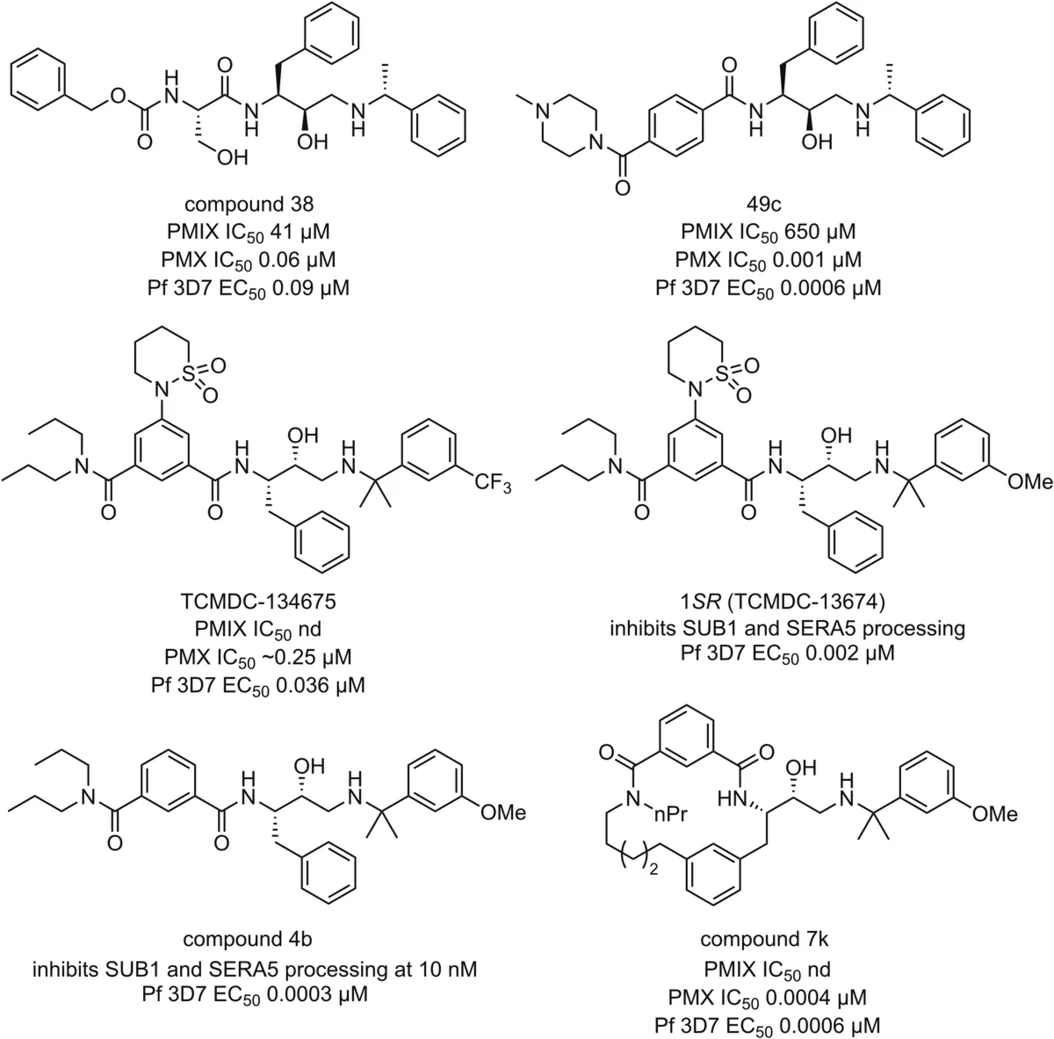
Structures and activity of PMX HEA inhibitors.

Mechanistic studies revealed that these compounds blocked SUB1 maturation and SERA5 processing, leading to reduced parasite viability between 40 and 48 h post‐invasion—phenotypes consistent with PMX knockdown [20, 21]. Microscopy confirmed that several compounds prevented merozoite egress, while knockdown assays demonstrated specificity for PMX, as SUB1 processing was abolished only in PMX‐depleted lines. Correspondingly, biochemical inhibition of PMX was closely aligned with parasite growth inhibition. For example, 49c displayed potent inhibition of PMX (IC_50_ 1 nM; Ki 0.19 nM), showed weaker inhibition of PMIX (IC_50_ 0.65 µM) (Figure 10), and potent anti‐plasmodial activity (EC_50_ 0.6 nM), indicating anti‐parasitic activity was primarily mediated through inhibition of PMX. Consistent with the established roles of PMX in activating SUB1 and cleaving invasion ligands (MSP1, SERA5, AMA1), and of PMIX in RAP1 and ASP processing, 49c blocked substrate cleavage of these proteins, thereby demonstrating parasite death through impaired egress and invasion.

Compound 49c was further evaluated in *P. berghei* murine and mosquito models to investigate the role of PMX in parasite survival [21]. In a 4‐day *P. berghei* asexual‐stage model, intraperitoneal administration at 100 mg/kg successfully cleared parasitemia and cured infected mice. In transmission‐stage assays, gametocytes matured to stage V but failed to produce functional gametes, suggesting that 49c may block gametocyte egress by inhibiting PMX‐dependent maturation of SUB1, although additional studies are required to confirm this mechanism. In mosquito models, 49c inhibited ookinete invasion, likely by preventing CelTOS processing, thereby implicating PMX in midgut penetration. During hepatic stages, sporozoite‐infected mice treated with 49c did not progress from liver schizonts to blood‐stage infection, despite intact merozoite morphology. Collectively, these findings suggest that PMX is a compelling multistage antimalarial target.

While compound 49c demonstrated promising efficacy in both murine and mosquito models, the search for inhibitors with improved drug‐like properties led to several independent programs identifying new starting points targeting PMX for antimalarial development. Among these, 1*SR* (TCMDC‐134674) and TCMDC‐134675 were discovered in a phenotypic screen of the TCAMS library against asexual‐stage parasites [154]. Both 1*SR* (TCMDC‐134674) and TCMDC‐134675 contain an HEA head group and share structural similarity to 49c, differing in substitutions at the C‐terminal benzylic and N‐terminal aryl carboxamide regions (Figure 10), which occupy the S_1_′ and S_2_ and S_3_ pockets of PMX, respectively [168]. The benzyl group of the HEA motif mimics the Phe in the P_1_ position of PMX (and PMIX) substrates [113, 117]. Optimization of this scaffold revealed that replacing the sultam on the aryl carboxamide with a hydrogen atom (compound 4b) enhanced anti‐parasitic activity (EC_50_ 0.3 nM) (Figure 10) and potently inhibited SUB1 and downstream SERA5 processing by PMX [168].

Structural analysis of compound 4b bound to PMX provided insights into further optimization that enabled further refinement, including macrocyclization linking the P_1_ benzyl group to the P_3_ aryl carboxamide motif, resulting in 7k (Figure 10). An X‐ray structure of 7k bound to PMX, in a partial closed flap configuration, confirmed the conformational orientation of this macrocyclic design (Figure 4D), which yielded enhanced PMX inhibition (IC_50_ 0.6 nM) and potent anti‐parasitic activity (EC_50_ 0.6 nM) [169, 170]. Importantly, 7k strongly inhibited SUB1 processing but not RAP1, indicating its activity was specific to PMX rather than PMIX. Pharmacokinetic profiling of 7k revealed moderate systemic exposure (*C*_max_ 0.064 µg/mL; AUC 13.1 µg/mL/min) and a half‐life of 53 min, supporting its suitability for twice‐ or thrice‐daily dosing to maintain plasma concentrations above the minimal parasitic threshold for 24 h. In a *P. falciparum* humanized mouse model, dosing at 40 mg/kg BID or TID reduced parasitemia to undetectable levels; however, recrudescence occurred upon cessation of treatment on Day 4. These findings highlight the need for further pharmacokinetic optimization of this compound class before advancing as a preclinical candidate.

Cyclic guanidine scaffolds, including the five‐membered amino hydantoin and the six‐membered amino (or imino) pyrimidinone, have historically been employed in drug discovery programs targeting BACE1 and renin for the treatment of Alzheimer's disease and hypertension. Among these, the amino hydantoins CWHM‐117 (8p) and TCMDC‐136879 demonstrated modest antiplasmodial activity (IC_50_ 0.46 and 2.76 µM) [159]. As outlined in Section 3.2, the anti‐parasitic activity was initially attributed to inhibition of PMII and PMIV but was later confirmed to result from inhibition of PMX (Figure 6) [21]. To date, no further optimization of the amino hydantoin scaffold has been reported, largely because research efforts have shifted toward more promising chemotypes such as the HEA and six‐membered amino (or imino) pyrimidinone (IP) classes.

A screen of a focused library identified an IP scaffold with potent PMX inhibition (IC_50_ 0.03 µM) and anti‐parasitic activity (EC_50_ 0.08 µM) [167]. Structural modifications to the pyran group occupying the S_2_′ pocket and inclusion of a P_1_ aryl amino group connected to a 4‐pyrimidine that extends into the S_3_ pocket of PMX yielded UCB7362 (Figure 4C). UCB7362 exhibited enhanced potency against PMX (IC_50_ 0.008 µM) and antiparasitic activity (EC_50_ 0.01 µM) (Figure 11), alongside > 500‐fold selectivity over human aspartyl proteases. UCB7362 engaged PMX in parasites and, to a lesser extent, PMIX, consistent with its modest inhibition of recombinant PMIX (IC_50_ 0.142 µM). Resistance profiling on UCB7362 revealed a moderate‐to‐high risk, with minimum inoculum of resistance (MIR) [11] values of 2 × 10^5^ and 9.6 × 10^6^. Genome sequencing of the recrudescent parasites identified a twofold to threefold amplification of the genome that encodes PMX, correlating with the twofold to threefold EC_50_‐shift with the resistant mutants. UCB7362 demonstrated high stability in human liver hepatocytes (CL_int_ 10 µL/min/10^6^ cells), modest stability in mouse liver hepatocytes (CL_int_ 42 µL/min/10^6^ cells), and high aqueous solubility in simulated intestinal fluid (> 1000 µM). Pharmacokinetic studies showed moderate clearance in rats (CL 44 mL/min/kg) but low clearance in beagles (CL 13 mL/min/kg) resulting in higher systemic exposure and oral bioavailability in beagles (*C*_max_ 4340 nM; AUC 34500 nM h; F 120%) compared with rats (*C*_max_ 246 nM; AUC 1410 nM h; V*ss* 5.7 L/kg; F 11%). In a *P. falciparum* humanized mouse model, oral administration of UCB7362 at 60 mg/kg b.i.d. for 4 days reduced parasitemia to undetectable levels, though recrudescence occurred after treatment cessation.

**Figure 11:**
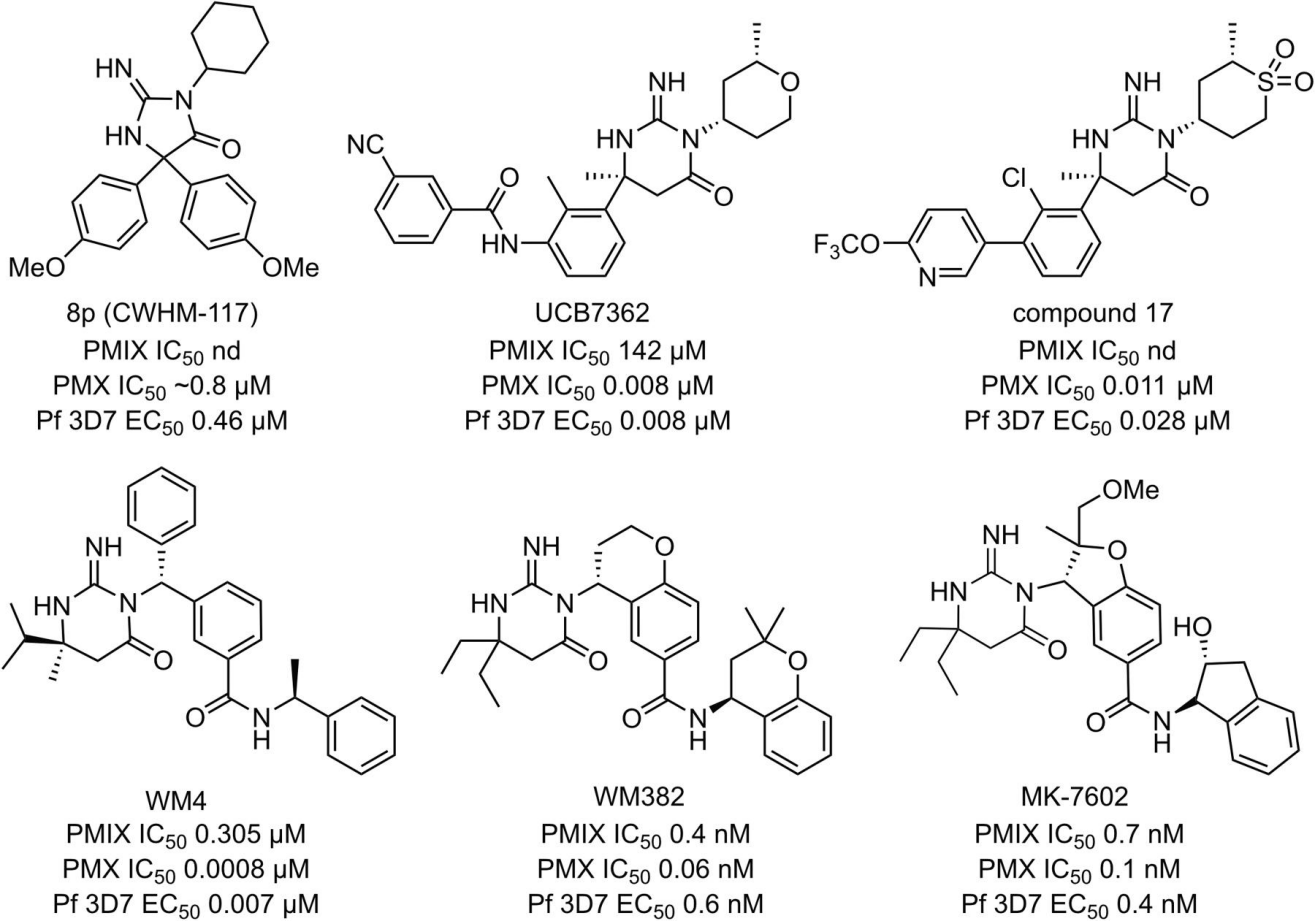
Structures and activity of dual PMIX and PMX cyclic guanidine inhibitors.

A close analog of UCB7362, compound 3, demonstrated improved efficacy in the same model but was limited by reduced safety margins [148]. To enhance pharmacokinetic parameters and mitigate safety concerns, modification to the S_1_′ pyran group and substitution of the S_3_ aryl group produced compound 17 (Figure 11) [171]. Compound 17 retained comparable PMX (IC_50_ 0.01 µM) and anti‐plasmodial potency (EC_50_ 0.03 µM) and selectivity against cathepsin D (140‐fold), while exhibiting improved pharmacokinetic properties in rats, including reduced clearance (CL 18 mL/min/kg), greater systemic exposure (*C*_max_ 423 nM; AUC 4680 nM h; V*ss* 13.1 L/kg) and enhanced bioavailability (F 29%). Further evaluation in mouse efficacy and safety models will be required to determine the suitability of compound 17 for preclinical development.

A phenotypic screen of the MSD library of aspartic protease inhibitors against the *P. falciparum* asexual stage parasite identified another class of IP compounds. Among the hits, WM4 exhibited potent anti‐plasmodial activity (EC_50_ 7 nM) (Figure 11) [108, 172]. Resistance selection with WM4 revealed genomic amplification of the PMX locus. Mechanistic studies showed that WM4 blocked SUB1 processing via SERA5 but did not affect ASP1 maturation, indicating that its antiparasitic activity was mediated through PMX rather than PMIX. On‐target activity was further confirmed by thermal stabilization of PMX in parasites, and biochemical assays demonstrated that WM4 potently inhibited PMX (IC_50_ 0.8 nM) while only modestly inhibiting PMIX (IC_50_ 305 nM).

Optimization of the WM4 scaffold involved substituting the isopropyl group with an ethyl group to better occupy the S_1_ pocket of PMX (and PMIX), inclusion of a P_1_′ ethyl group, constraining the benzylic linker with a chromane via the S_2_ pocket, and replacing the carboxamide aryl group with a 2,2‐dimethyl chromane moiety to better occupy the S_3_ pocket which has an extended loop motif (Figure 4A,B). These modifications culminated in WM382 (Figure 11) [113, 172]. WM382 exhibited highly potent dual inhibition of PMIX and PMX (IC_50_ 0.4 and 0.06 nM), and potent anti‐parasitic activity (EC_50_ 0.6 nM). WM382 potently blocked the maturation of multiple PMIX substrates, including ASP, RON3 and RAP1 and PMX substrates, including SUB1, SUB2, Rh1, and Rh4 (Table 2). The dual inhibition of these essential proteases imposed a substantial barrier to resistance, as such resistant parasites could not be selected with WM382 [173].

Pharmacokinetic profiling of WM382 in rats revealed moderate clearance, good systemic exposure, and oral bioavailability (CL_p_ 46 mL/min/kg; *t*_1/2_ 5.9 h; V*ss* 9.2 L/kg; AUC 2.6 µM.h; F% 21). In vivo, four once‐daily oral doses of WM382 at 30 mg/kg cured mice of *P. berghei* infection, while 3 mg/kg reduced parasitemia to undetectable levels in *P. falciparum* humanized mice [108]. WM382 also blocked transmission at 2.5 nM by preventing parasite transfer from infected blood to mosquitoes, and two oral doses at 20 mg/kg during liver‐stage development prevented establishment of blood‐stage infection, highlighting its potential as a chemopreventative or transmission‐blocking therapy. Furthermore, as noted in Section 2.7, *P. berghei* infected mice treated with WM382 could not be reinfected [109], suggesting a potential application in a chemo‐vaccination strategy.

Further optimization of the WM382 scaffold to enhance pharmacokinetics and safety margins led to the development of MK‐7602 (Figure 11) [126]. This was achieved by modifying the chromane moiety to a 2,2‐disubstituted dihydrobenzofuran and replacing the dimethyl chromane with a 3‐hydroxyl‐substituted indane. Structurally, the dihydrobenzofuran ring system interacts with amino acids in the S_2_′ pocket while the hydroxyl group on the indane interacts with S211 in the S_3_ pocket of PMX and PMIX (Figure 4B). These modifications conferred potent inhibition of both PMIX and PMX (*K_i_* 0.68 and 0.13 nM), with > 4000‐fold selectivity over human aspartyl proteases and potent anti‐plasmodial activity (EC_50_ 0.40 nM). Thermal profiling proteomics and western blot analyses confirmed that MK‐7602 engages both PMIX and PMX in parasites, effectively blocking processing of their substrates [126]. In the MIR platform, recrudescence was not observed even at the highest inoculum (MIR > 10^9^), whereas resistance emerged after 15 months of continuous selection with incremental increases of MK‐7602 [126, 173]. The resistant parasites displayed amplification of the genomic regions encoding PMIX and PMX. Notably, in the absence of MK‐7602, these mutants rapidly reverted to wild‐type, highlighting the substantial fitness cost associated with gene amplification. Collectively, these findings demonstrate that MK‐7602 possesses a remarkably high barrier to the development of resistance.

The pharmacokinetic profile of MK‐7602 was broadly comparable to that of WM382, characterized in rats by moderate clearance, good systemic exposure, and oral bioavailability (CL_p_ 60 mL/min/kg; *t*_1/2_ 5.9 h; *V*_d*ss*_ 9.7 L/kg; F% 26) [126]. Notably, MK‐7602 demonstrated improved properties in dogs (CL_p_ 8.9 mL/min/kg; *t*_1/2_ 4.1 h; V_d*ss*_ 4.1 L/kg; F% 66) and favorable predicted pharmacokinetics in humans (CL 3.93 mL/min/kg; terminal *t*_1/2_ 23.5 h; *V*_d*ss*_ 4.1 L/kg; F% 38). In addition, MK‐7602 exhibited a superior safety profile compared with WM382, particularly with respect to hERG cardiac ion channel inhibition, showing a 40‐fold improvement. In efficacy studies, oral twice‐daily dosing of MK‐7602 at 50 mg/kg cured mice infected with *P. berghei* asexual‐stage parasites. In liver‐stage infection models, twice‐daily treatment at 100 mg/kg significantly reduced the establishment of subsequent asexual blood‐stage infection. Transmission profiling revealed that MK‐7602 did not lower parasitemia during gametocytogenesis; however, the treated gametocytes were defective and failed to form oocysts in the mosquito midgut. Consistent with this, *P. berghei*‐infected mice treated with 20 mg/kg MK‐7602 prevented transmission to mosquitoes following a blood meal. Together, these findings highlight the promising preclinical safety and efficacy profile of MK‐7602, which ultimately supported its advancement into clinical development.

MK‐7602 was evaluated in a phase Ia clinical trial (NCT06797674) to assess tolerability and pharmacokinetics following single and multiple ascending oral doses [174, 175]. MK‐7602 was well tolerated at doses of up to 400 mg. Pharmacokinetic analysis of a single oral dose revealed a *T*_max_ of 1.8–2.5 h and a half‐life of 23.2–24.2 h, with plasma concentrations exceeding five times the EC_50_ minimum parasiticidal concentration (MPC) at a 100 mg dose. With repeated daily dosing, the half‐life was extended to 34–52 h. Subsequently, MK‐7602 was evaluated in a phase 1b Controlled Human Malaria Infection (CHMI) study in healthy volunteers (NCT06294912) [174]. A single 400 mg oral dose rapidly reduced parasitemia to below detectable levels, with a parasite clearance half‐life of 3.84 h, although recrudescence occurred 4 days post‐treatment. In contrast, a regimen of 200 mg daily for 3 days achieved complete clearance with a parasite clearance half‐life of 4.55 h, and no recrudescence of asexual parasitemia was observed. Notably, while significant gametocyte titers were detected following clearance of asexual parasites, preclinical data indicate that MK‐7602‐treated gametocytes are non‐viable and unable to form oocysts in the mosquito midgut, suggesting they may not be transmissible. Collectively, these phase I clinical findings demonstrate encouraging safety, pharmacokinetics, and efficacy, supporting the continued clinical development of MK‐7602 as a candidate for the treatment of *P. falciparum* malaria.

## 4 Conclusions and Outlook

Since the discovery of aspartyl proteases in the digestive vacuole [28] these enzymes have attracted considerable interest as potential drug targets for antimalarial therapy. However, despite extensive efforts, no antimalarial agents emerged from this line of investigation, largely because the essentiality of PMs I–IV in parasite survival was not clearly established, an issue compounded by the limited experimental tools available at the time. This highlights the importance of integrating genetic, cell biological, and proteomic approaches to rigorously define both the essentiality and druggability of candidate molecular targets. Moreover, a precise understanding of how small molecules interact with their targets and how this translates into antiparasitic activity is critical for successful drug development. Ultimately, digestive vacuole PMs I–IV have been excluded as viable antimalarial targets due to their functional redundancy in parasite survival.

PMVII appears to be dispensable for *P. berghei* parasite development [106], although its role has not yet been investigated in human‐infectious *Plasmodium* species. Additional tools are needed to clarify the essential functions of PMVI and PMVIII during parasite development in the mosquito [100, 107] and to assess their potential as transmission‐blocking targets. Identification of the natural substrates of PMVI and PMVIII would provide critical insight into their precise biological roles and enable the design of peptidomimetics as probes for functional studies. Moreover, recombinant expression of these proteases would facilitate direct screening of existing aspartyl protease inhibitors as potential starting points for selective inhibitor development. Consideration of compound stability within mosquitoes will be essential if such inhibitors are to be incorporated into mass‐administered therapies in malaria‐endemic regions to block transmission. Ultimately, PMVI and PMVIII inhibitors may also find application in sugar bait as a strategy to interrupt transmission in endemic villages.

Recent drug discovery efforts have been directed towards uncovering small molecule inhibitors of either PMV or PMIX and PMX. PMV is essential for both asexual and sexual development [72, 79, 81], and there is increasing evidence supporting the vital role of PMV in liver stage development [90, 95]. Current strategies to target PMV have primarily relied on peptidomimetic probes, which have proven valuable for exploring parasite biology and validating PMV as an antimalarial target [73, 74, 75, 77, 78]. Challenges remain with uncovering suitable drug‐like starting points to target PMV. This restraint has largely stemmed from the unique substrate specificity profile of PMV that is distinct from any human aspartyl protease. As a result, inhibitors of human aspartyl proteases display weak activity against PMV and, to date, have proven recalcitrant to optimization for this target. Compounding this challenge, PMV is highly abundant during the asexual blood stage, and parasite growth is only modestly affected even when > 95% of PMV is deleted [80]. Consequently, inhibitors must achieve exceptionally high potency and be administered at concentrations far exceeding the biochemical IC_50_ to demonstrate efficacy. To date, no tractable drug‐like scaffolds have been identified that potently inhibit PMV, representing a significant obstacle to the development of PMV‐targeted antimalarials.

PMIX and PMX are both indispensable for asexual stage development and are bona fide antimalarial drug targets [20, 21, 108]. In addition, PMX is required for successful transmission to the mosquito vector and for progression from the liver stage to the asexual blood stage, making it an attractive target for prophylactic or transmission‐blocking therapies [21, 108, 126]. The high degree of homology in the ligand‐binding clefts of PMIX and PMX [113] supports dual inhibition of both of these aspartyl proteases, and indeed inhibitors such as WM382 and MK‐7602 [108, 126, 172], have demonstrated exceptionally high barriers to resistance [173], exemplifying the appeal of simultaneously targeting both proteases. Although relatively selective inhibitors of PMX have been discovered [108, 167, 168, 170, 172, 173], a selective PMIX inhibitor has remained elusive. An X‐ray structure of PMIX could inform design of a selective inhibitor, and as AI modeling technologies become more reliable and commonplace, they could also assist with selective inhibitor design. Such compounds would be valuable for probing and delineating the distinct biological roles and substrate specificities of PMIX and PMX. Progress in targeting PMIX and PMX has been facilitated by their similarities to human aspartyl proteases, which provide drug‐like starting points with high potency. Importantly, discrete differences in amino acid composition in ligand binding pockets of PMIX and PMX compared with human aspartyl proteases enabled fine‐tuning of selectivity, ensuring suitable safety margins for dosing in humans. UCB7362 and MK‐7602 exemplify this strategy [126, 167], with MK‐7602 advancing to human clinical development and completing phase I clinical trials (NCT06797674, NCT06294912) [174]. The outcomes of subsequent clinical studies on MK‐7602 are eagerly anticipated.

Beyond treatment, PMIX/PMX‐targeted inhibitors may also be deployed in seasonal chemopreventative regimens. The design of a highly potent PMIX/PMX inhibitor with a long systemic half‐life for administration in an LAI formulation is pertinent for this approach. Chemovaccination represents another exciting prospect, whereby PMIX/PMX‐targeted therapies could confer protective immunity across subsequent transmission seasons [109]. The mechanisms underpinning the protective immunity and breadth of the protection afforded will be the subject of intense research and high interest in the field. Collectively, PMIX and PMX inhibitors hold transformative potential for both malaria prevention and treatment, adding to the growing arsenal of promising antimalarial candidates in clinical development.

Insights gained from studying PMs can be extended to related Apicomplexan parasites, offering opportunities to design protease‐targeted therapies for diseases of major human and animal health importance. Aspartyl proteases are widely expressed across the Apicomplexa phylum, with emerging evidence indicating that several play critical roles in parasite survival. In *Toxoplasma gondii*, the causative parasite of toxoplasmosis in humans, seven cathepsin‐D like aspartyl proteases (TgASP1 to TgASP7) are encoded, although only TgASP1, TgASP3 and TgASP5 are expressed during the tachyzoite stage [18]. TgASP1, the ortholog of *P. falciparum* PM I–IV, localizes to the lysosome‐like vacuolar compartment but is dispensable for lysosomal protein digestion [176] and tachyzoite development [177]. TgASP3, phylogenetically related to PMIX and PMX, plays a critical role in the maturation of ligands required for parasite invasion and egress from the host cell [178]. TgASP3 has the same substrate consensus sequence as PMX (SF*hQ/E/D) and is inhibited by the tool compound 49c [179]. TgASP5 functions analogously to its *Plasmodium* ortholog PMV, mediating protein export into the host cell [180, 181, 182]. The peptidomimetic compound WEHI‐586, which mimics the RRL motif of the *Toxoplasma* export element (TEXEL), potently inhibits TgASP5 in biochemical assays and represents a valuable probe for future studies of ASP5 biology [180].

Parasites of the genus *Cryptosporidium* are a major global health burden, causing diarrheal disease in humans and other mammals. Research on *Cryptosporidium* has historically lagged behind that of *Plasmodium* and *Toxoplasma*, largely due to technical challenges in studying the parasite. Quite recently, genetic manipulation techniques have emerged [183], enabling new insights into the role of proteins, including aspartyl proteases, in disease pathogenesis. The *Cryptosporidium* genome encodes five aspartyl proteases, CpASP1‐5 (cgd1_2240; CpASP1, cgd1_3690; CpASP2, cgd4_2190; CpASP3, cgd6_660; CpASP4, and cgd6_3820; CpASP5) [184]. CpASP1 and CpASP2 are both expressed in both asexual and sexual stages, though CpASP1 is dispensable for parasite development. In contrast, CpASP2 is refractory to deletion and is required for asexual and male gamete egress from host enterocytes [184]. Interestingly, sequence and phylogenetic analyses place CpASP2 closest to PMV, yet its functional role in parasite egress and development aligns more closely with PMX. Exported *Cryptosporidium* proteins, such as mucin‐like enhanced by differentiation lectin‐like extracellular exported 2 protein (MEDLE2) and microvilli protein 1 (MVP1) contain an N‐terminal SF*h motif resembling the PMX cleavage sequence [185, 186], suggesting parallels in function and substrate recognition. Preliminary evidence indicates that CpASP4 may process exported effector proteins [187], although further studies are needed to confirm whether its role mirrors that of PMV in *Plasmodium* parasites.

*Babesia* parasites, the causative organism of babesiosis in livestock, express four cathepsin‐D like aspartyl proteases (ASP2, 3, 5, 6) [188]. Little is known about ASP5 and ASP6. ASP5 belongs to the same clade D as PMV and is therefore believed to perform the same essential role in protein export beyond the parasite membrane. Supporting this notion, *Babesia* parasites export a myriad of proteins with a PEXEL‐like motif [189, 190], and some of these have been characterized to play important roles in red blood cell adhesion and immune evasion [190, 191]. ASP6 belongs to clade B and is thought to be expressed in the tick, and from a phylogeny context, putatively may have similar roles to PMs IV‐III in the mosquito [188]. Moreover, further investigation into ASP5 and ASP6 in *Babesia* parasites is required to qualify their potential roles for validation as drug targets. Most recently, *Babesia divergens* ASP2 and ASP3 were shown to recapitulate the essential roles of PMIX and PMX in *Plasmodium* [192]*. B. divergens* parasites deficient in ASP3 could not invade or egress from the host red blood cell, while parasites lacking ASP2 had a defect in invasion. Accordingly, ASP3 was localized to the micronemes and ASP2 to the rhoptry bulb. Notably, WM382 (a dual PMIX and PMX inhibitor) exhibited synergistic activity with an ASP2 and an ASP3 knockdown, while UCB7362 (a PMX‐specific inhibitor) only showed synergistic activity with an ASP2 knockdown in *B. divergens* parasites [192]. This suggests that further optimization of these compounds against either recombinant ASP2 and ASP3 or against *Babesia* parasites could be a promising avenue to treat babesiosis in agricultural settings.

Other apicomplexan parasites, including *Theileria*, *Neospora*, and *Eimeria*, also encode cathepsin D‐like aspartyl proteases [193]. Phylogenetic analyses indicate that *Eimeria* species express two related aspartyl proteases, termed eimepsins. Eimepsin 1 belongs to clade B and shares homology with plasmepsins PMVI and PMVIII, whereas eimepsin 3 clusters within clade C alongside PMIX and PMX. *Theileria* species encode multiple aspartyl proteases, including ASP2 (clade B), ASP3 (clade C), ASP5 (clade D), and ASP6 (clade E), while *Neospora* species express ASP2 (clade B), ASP3 (clade C), ASP4 (clade B), and ASP5 (clade D) [193]. Little is known about the expression, localization, essentiality, or function of these proteases. Nevertheless, their phylogenetic relationships suggest conserved roles in parasite survival and biology. Consequently, further characterization of these aspartyl proteases is warranted, particularly given their potential as novel drug targets for the control of agriculturally important apicomplexan pathogens. Collectively, these observations suggest that aspartyl proteases across the Apicomplexa phylum share functional similarities with *Plasmodium* PMs, underscoring opportunities to repurpose existing aspartyl protease inhibitors for the treatment of apicomplexan diseases in both humans and animals.

The high degree of homology observed among certain aspartyl proteases across Apicomplexa species raises the possibility of designing cross‐species inhibitors, an approach that could be attractive from a cost and development perspective. However, such a “silver‐bullet” strategy would likely pose significant challenges, including the emergence of resistance resulting from widespread use, paralleling lessons learned from the broad application of antibiotics and other anti‐infective agents. Effective inhibitors would require distinct pharmacokinetic profiles tailored to species‐specific parasite lifecycles and tissue tropisms. For example, inhibitors targeting *Cryptosporidium* infections would ideally be gut‐restricted, whereas those directed against blood‐stage parasites would require sustained systemic exposure. Differences in compliance and cost‐effectiveness between animal and human therapeutics further complicate this approach, particularly in low‐resource regions that bear the greatest burden of apicomplexan diseases. For these reasons, the development of species‐specific aspartyl protease inhibitors is strongly advocated, particularly those that simultaneously target two essential enzymes within a single species to mitigate resistance, as exemplified by dual PMIX and PMX inhibitors currently in clinical development [108, 126, 173]. Not only will such targeted inhibitors provide valuable tools to elucidate the roles of aspartyl proteases in Apicomplexa biology, but they also represent a critical unmet need in the prevention and treatment of apicomplexan diseases. The progression of the first‐in‐class plasmepsin inhibitor MK‐7602 into human clinical trials for malaria demonstrates the promise of this targeted strategy and supports its extension to other apicomplexan infections.

## Data Availability

Data sharing is not applicable to this article as no data sets were generated or analyzed during the current study.

## References

[1] WHO , World Malaria Report, World Health Organisation, Geneva, accessed December 23, 2025, https://www.who.int/teams/global‐malaria‐programme/reports/world‐malaria‐report‐2025.

[2] K. Mendis , B. Sina , P. Marchesini , and R. Carter , “The Neglected Burden of *Plasmodium vivax* Malaria,” supplement, American Journal of Tropical Medicine and Hygiene 64, no. 1–2 Suppl (2001): 97–106. 10.4269/ajtmh.2001.64.97 11425182

[3] C. J. Sutherland , “Persistent Parasitism: The Adaptive Biology of *malariae* and *ovale* Malaria,” Trends in Parasitology 32, no. 10 (2016): 808–819. 10.1016/j.pt.2016.07.001 27480365

[4] B. Singh , L. K. Sung , A. Matusop , et al., “A Large Focus of Naturally Acquired *Plasmodium knowlesi* Infections in Human Beings,” Lancet 363, no. 9414 (2004): 1017–1024. 10.1016/S0140-6736(04)15836-4 15051281

[5] P. E. Duffy , J. P. Gorres , S. A. Healy , and M. Fried , “Malaria Vaccines: A New Era of Prevention and Control,” Nature Reviews Microbiology 22, no. 12 (2024): 756–772. 10.1038/s41579-024-01065-7 39025972

[6] M. S. Datoo , A. Dicko , H. Tinto , et al., “Safety and Efficacy of Malaria Vaccine Candidate R21/Matrix‐M in African Children: A Multicentre, Double‐Blind, Randomised, Phase 3 Trial,” Lancet 403, no. 10426 (2024): 533–544. 10.1016/S0140-6736(23)02511-4 PMC7618965 38310910

[7] K. Haldar , S. Bhattacharjee , and I. Safeukui , “Drug Resistance in *Plasmodium* ,” Nature Reviews Microbiology 16, no. 3 (2018): 156–170. 10.1038/nrmicro.2017.161 PMC6371404 29355852

[8] E. A. Ashley , M. Dhorda , R. M. Fairhurst , et al., “Spread of Artemisinin Resistance in *Plasmodium falciparum* Malaria,” New England Journal of Medicine 371, no. 5 (2014): 411–423. 10.1056/NEJMoa1314981 PMC4143591 25075834

[9] A. Uwimana , E. Legrand , B. H. Stokes , et al., “Emergence and Clonal Expansion of In Vitro Artemisinin‐Resistant *Plasmodium falciparum* kelch13 R561H Mutant Parasites in Rwanda,” Nature Medicine 26, no. 10 (2020): 1602–1608. 10.1038/s41591-020-1005-2 PMC7541349 32747827

[10] P. J. Rosenthal , V. Asua , J. A. Bailey , et al., “The Emergence of Artemisinin Partial Resistance in Africa: How Do We Respond?,” Lancet Infectious Diseases 24, no. 9 (2024): e591–e600. 10.1016/S1473-3099(24)00141-5 PMC12954456 38552654

[11] M. Duffey , B. Blasco , J. N. Burrows , T. N. C. Wells , D. A. Fidock , and D. Leroy , “Assessing Risks of *Plasmodium falciparum* Resistance to Select Next‐Generation Antimalarials,” Trends In Parasitology 37, no. 8 (2021): 709–721. 10.1016/j.pt.2021.04.006 PMC8282644 34001441

[12] J. N. Burrows , S. Duparc , W. E. Gutteridge , et al., “New Developments in Anti‐Malarial Target Candidate and Product Profiles,” Malaria Journal 16, no. 1 (2017): 26. 10.1186/s12936-016-1675-x PMC5237200 28086874

[13] T. D. Ashton , S. M. Devine , J. J. Möhrle , et al., “The Development Process for Discovery and Clinical Advancement of Modern Antimalarials,” Journal of Medicinal Chemistry 62, no. 23 (2019): 10526–10562. 10.1021/acs.jmedchem.9b00761 31385706

[14] J. L. Siqueira‐Neto , K. J. Wicht , K. Chibale , J. N. Burrows , D. A. Fidock , and E. A. Winzeler , “Antimalarial Drug Discovery: Progress and Approaches,” Nature Reviews Drug Discovery 22, no. 10 (2023): 807–826. 10.1038/s41573-023-00772-9 PMC10543600 37652975

[15] J. Okombo and D. A. Fidock , “Towards Next‐Generation Treatment Options to Combat *Plasmodium falciparum* Malaria,” Nature Reviews Microbiology 23, no. 3 (2025): 178–191. 10.1038/s41579-024-01099-x PMC11832322 39367132

[16] M. J. Gardner , N. Hall , E. Fung , et al., “Genome Sequence of the Human Malaria Parasite *Plasmodium falciparum* ,” Nature 419, no. 6906 (2002): 498–511. 10.1038/nature01097 PMC3836256 12368864

[17] M. Zhang , C. Wang , T. D. Otto , et al., “Uncovering the Essential Genes of the Human Malaria Parasite *Plasmodium falciparum* by Saturation Mutagenesis,” Science 360, no. 6388 (2018): eaap7847. 10.1126/science.aap7847 PMC6360947 29724925

[18] M. Shea , U. Jäkle , Q. Liu , C. Berry , K. A. Joiner , and D. Soldati‐Favre , “A Family of Aspartic Proteases and a Novel, Dynamic and Cell‐Cycle‐Dependent Protease Localization in the Secretory Pathway of *Toxoplasma gondii* ,” Traffic 8, no. 8 (2007): 1018–1034. 10.1111/j.1600-0854.2007.00589.x 17547703

[19] J. Liu , I. Y. Gluzman , M. E. Drew , and D. E. Goldberg , “The Role of *Plasmodium falciparum* Food Vacuole Plasmepsins,” Journal of Biological Chemistry 280, no. 2 (2005): 1432–1437. 10.1074/jbc.M409740200 15513918

[20] A. S. Nasamu , S. Glushakova , I. Russo , et al., “Plasmepsins IX and X Are Essential and Druggable Mediators of Malaria Parasite Egress and Invasion,” Science 358, no. 6362 (2017): 518–522. 10.1126/science.aan1478 PMC5928414 29074774

[21] P. Pino , R. Caldelari , B. Mukherjee , et al., “A Multistage Antimalarial Targets the Plasmepsins IX and X Essential for Invasion and Egress,” Science 358, no. 6362 (2017): 522–528. 10.1126/science.aaf8675 PMC5730047 29074775

[22] T. Weißbach , A. Golzmann , S. Bennink , G. Pradel , and C. Julius Ngwa , “Transcript and Protein Expression Analysis of Proteases in the Blood Stages of *Plasmodium falciparum* ,” supplement, Experimental Parasitology 180, no. Suppl C (2017): 33–44. 10.1016/j.exppara.2017.03.006 28351685

[23] M. Klemba and D. E. Goldberg , “Characterization of Plasmepsin V, a Membrane‐Bound Aspartic Protease Homolog in the Endoplasmic Reticulum of *Plasmodium falciparum* ,” Molecular and Biochemical Parasitology 143, no. 2 (2005): 183–191. 10.1016/j.molbiopara.2005.05.015 16024107

[24] J. A. Boddey , A. N. Hodder , S. Günther , et al., “An Aspartyl Protease Directs Malaria Effector Proteins to the Host Cell,” Nature 463, no. 7281 (2010): 627–631. 10.1038/nature08728 PMC2818761 20130643

[25] I. Russo , S. Babbitt , V. Muralidharan , T. Butler , A. Oksman , and D. E. Goldberg , “Plasmepsin V Licenses *Plasmodium* Proteins for Export Into the Host Erythrocyte,” Nature 463, no. 7281 (2010): 632–636. 10.1038/nature08726 PMC2826791 20130644

[26] P. M. Cheuka , G. Dziwornu , J. Okombo , and K. Chibale , “Plasmepsin Inhibitors in Antimalarial Drug Discovery: Medicinal Chemistry and Target Validation (2000 to Present),” Journal of Medicinal Chemistry 63, no. 9 (2020): 4445–4467. 10.1021/acs.jmedchem.9b01622 31913032

[27] A. S. Nasamu , A. J. Polino , E. S. Istvan , and D. E. Goldberg , “Malaria Parasite Plasmepsins: More Than Just Plain Old Degradative Pepsins,” Journal of Biological Chemistry 295, no. 25 (2020): 8425–8441. 10.1074/jbc.REV120.009309 PMC7307202 32366462

[28] D. E. Goldberg , A. F. Slater , R. Beavis , B. Chait , A. Cerami , and G. B. Henderson , “Hemoglobin Degradation in the Human Malaria Pathogen *Plasmodium falciparum*: A Catabolic Pathway Initiated by a Specific Aspartic Protease,” Journal of Experimental Medicine 173, no. 4 (1991): 961–969. 10.1084/jem.173.4.961 PMC2190804 2007860

[29] D. L. vander Jagt , L. A. Hunsaker , N. M. Campos , and J. V. Scaletti , “Localization and Characterization of Hemoglobin‐Degrading Aspartic Proteinases From the Malarial Parasite *Plasmodium falciparum* ,” Biochimica et Biophysica Acta (BBA)—Protein Structure and Molecular Enzymology 1122, no. 3 (1992): 256–264. 10.1016/0167-4838(92)90401-x 1504087

[30] K. E. Luker , S. E. Francis , I. Y. Gluzman , and D. E. Goldberg , “Kinetic Analysis of Plasmepsins I and II Aspartic Proteases of the *Plasmodium falciparum* Digestive Vacuole,” Molecular and Biochemical Parasitology 79, no. 1 (1996): 71–78. 10.1016/0166-6851(96)02651-5 8844673

[31] D. M. Wyatt and C. Berry , “Activity and Inhibition of Plasmepsin IV, A New Aspartic Proteinase From the Malaria Parasite, *Plasmodium falciparum* ,” FEBS Letters 513, no. 2–3 (2002): 159–162. 10.1016/s0014-5793(02)02241-x 11904142

[32] R. Banerjee , J. Liu , W. Beatty , L. Pelosof , M. Klemba , and D. E. Goldberg , “Four Plasmepsins Are Active in the *Plasmodium falciparum* Food Vacuole, Including a Protease With an Active‐Site Histidine,” Proceedings of the National Academy of Sciences 99, no. 2 (2002): 990–995. 10.1073/pnas.022630099 PMC117418 11782538

[33] M. E. Drew , R. Banerjee , E. W. Uffman , S. Gilbertson , P. J. Rosenthal , and D. E. Goldberg , “ *Plasmodium* Food Vacuole Plasmepsins Are Activated by Falcipains,” Journal of Biological Chemistry 283, no. 19 (2008): 12870–12876. 10.1074/jbc.M708949200 PMC2442342 18308731

[34] V. Mishra , I. Rathore , A. Arekar , et al., “Deciphering the Mechanism of Potent Peptidomimetic Inhibitors Targeting Plasmepsins—Biochemical and Structural Insights,” FEBS Journal 285, no. 16 (2018): 3077–3096. 10.1111/febs.14598 PMC6309945 29943906

[35] K. Jaudzems , K. Tars , G. Maurops , et al., “Plasmepsin Inhibitory Activity and Structure‐Guided Optimization of a Potent Hydroxyethylamine‐Based Antimalarial Hit,” ACS Medicinal Chemistry Letters 5, no. 4 (2014): 373–377. 10.1021/ml4004952 PMC4027636 24900843

[36] S. E. Francis , R. Banerjee , and D. E. Goldberg , “Biosynthesis and Maturation of the Malaria Aspartic Hemoglobinases Plasmepsins I and II,” Journal of Biological Chemistry 272, no. 23 (1997): 14961–14968. 10.1074/jbc.272.23.14961 9169469

[37] J. Hill , L. Tyas , L. H. Phylip , J. Kay , B. M. Dunn , and C. Berry , “High Level Expression and Characterisation of Plasmepsin II, an Aspartic Proteinase From *Plasmodium falciparum* ,” FEBS Letters 352, no. 2 (1994): 155–158. 10.1016/0014-5793(94)00940-6 7925966

[38] R. P. Moon , L. Tyas , U. Certa , et al., “Expression and Characterisation of Plasmepsin I From *Plasmodium falciparum* ,” European Journal of Biochemistry 244, no. 2 (1997): 552–560. 10.1111/j.1432-1033.1997.00552.x 9119023

[39] R. P. Moon , D. Bur , H. Loetscher , et al., “Studies on Plasmepsins I and II From the Malarial Parasite *Plasmodium falciparum* and Their Exploitation as Drug Targets,” Advances in Experimental Medicine and Biology 436 (1998): 397–406. 10.1007/978-1-4615-5373-1_56 9561248

[40] C. Boss , S. Richard‐Bildstein , T. Weller , W. Fischli , S. Meyer , and C. Binkert , “Inhibitors of the *Plasmodium falciparum* Parasite Aspartic Protease Plasmepsin II as Potential Antimalarial Agents,” Current Medicinal Chemistry 10, no. 11 (2003): 883–907. 10.2174/0929867033457674 12678679

[41] K. Ersmark , B. Samuelsson , and A. Hallberg , “Plasmepsins as Potential Targets for New Antimalarial Therapy,” Medicinal Research Reviews 26, no. 5 (2006): 626–666. 10.1002/med.20082 16838300

[42] A. P. Huizing , M. Mondal , and A. K. H. Hirsch , “Fighting Malaria: Structure‐Guided Discovery of Nonpeptidomimetic Plasmepsin Inhibitors,” Journal of Medicinal Chemistry 58, no. 13 (2015): 5151–5163. 10.1021/jm5014133 25719272

[43] M. J. Meyers and D. E. Goldberg , “Recent Advances in Plasmepsin Medicinal Chemistry and Implications for Future Antimalarial Drug Discovery Efforts,” Current Topics in Medicinal Chemistry 12, no. 5 (2012): 445–455. 10.2174/156802612799362959 PMC11670882 22242846

[44] B. S. Crabb , B. M. Cooke , J. C. Reeder , et al., “Targeted Gene Disruption Shows That Knobs Enable Malaria‐Infected Red Cells to Cytoadhere Under Physiological Shear Stress,” Cell 89, no. 2 (1997): 287–296. 10.1016/s0092-8674(00)80207-x 9108483

[45] A. L. Omara‐Opyene , P. A. Moura , C. R. Sulsona , et al., “Genetic Disruption of the *Plasmodium falciparum* Digestive Vacuole Plasmepsins Demonstrates Their Functional Redundancy,” Journal of Biological Chemistry 279, no. 52 (2004): 54088–54096. 10.1074/jbc.M409605200 15491999

[46] J. A. Bonilla , P. A. Moura , T. D. Bonilla , C. A. Yowell , D. A. Fidock , and J. B. Dame , “Effects on Growth, Hemoglobin Metabolism and Paralogous Gene Expression Resulting From Disruption of Genes Encoding the Digestive Vacuole Plasmepsins of *Plasmodium falciparum* ,” International Journal for Parasitology 37, no. 3–4 (2007): 317–327. 10.1016/j.ijpara.2006.11.008 17207486

[47] J. A. Bonilla , T. D. Bonilla , C. A. Yowell , H. Fujioka , and J. B. Dame , “Critical Roles for the Digestive Vacuole Plasmepsins of *Plasmodium falciparum* in Vacuolar Function,” Molecular Microbiology 65, no. 1 (2007): 64–75. 10.1111/j.1365-2958.2007.05768.x 17581121

[48] J. Liu , E. S. Istvan , I. Y. Gluzman , J. Gross , and D. E. Goldberg , “ *Plasmodium falciparum* Ensures Its Amino Acid Supply With Multiple Acquisition Pathways and Redundant Proteolytic Enzyme Systems,” Proceedings of the National Academy of Sciences 103, no. 23 (2006): 8840–8845. 10.1073/pnas.0601876103 PMC1470969 16731623

[49] J. B. Dame , C. A. Yowell , L. Omara‐Opyene , J. M. Carlton , R. A. Cooper , and T. Li , “Plasmepsin 4, the Food Vacuole Aspartic Proteinase Found in All *Plasmodium spp*. Infecting Man,” Molecular and Biochemical Parasitology 130, no. 1 (2003): 1–12. 10.1016/s0166-6851(03)00137-3 14550891

[50] J. Lin , R. Spaccapelo , E. Schwarzer , et al., “Replication of *Plasmodium* in Reticulocytes Can Occur Without Hemozoin Formation, Resulting in Chloroquine Resistance,” Journal of Experimental Medicine 212, no. 6 (2015): 893–903. 10.1084/jem.20141731 PMC4451122 25941254

[51] R. Spaccapelo , C. J. Janse , S. Caterbi , et al., “Plasmepsin 4‐Deficient *Plasmodium berghei* Are Virulence Attenuated and Induce Protective Immunity Against Experimental Malaria,” American Journal of Pathology 176, no. 1 (2010): 205–217. 10.2353/ajpath.2010.090504 PMC2797883 20019192

[52] R. Spaccapelo , E. Aime , S. Caterbi , et al., “Disruption of Plasmepsin‐4 and Merozoites Surface Protein‐7 Genes in *Plasmodium berghei* Induces Combined Virulence‐Attenuated Phenotype,” Scientific Reports 1 (2011): 39. 10.1038/srep00039 PMC3216526 22355558

[53] J. A. Boddey , T. G. Carvalho , A. N. Hodder , et al., “Role of Plasmepsin V in Export of Diverse Protein Families From the *Plasmodium falciparum* Exportome,” Traffic 14, no. 5 (2013): 532–550. 10.1111/tra.12053 23387285

[54] A. Heiber , F. Kruse , C. Pick , et al., “Identification of New PNEPs Indicates a Substantial Non‐PEXEL Exportome and Underpins Common Features in *Plasmodium falciparum* Protein Export,” PLoS Pathogens 9, no. 8 (2013): e1003546. 10.1371/journal.ppat.1003546 PMC3738491 23950716

[55] T. J. Sargeant , M. Marti , E. Caler , et al., “Lineage‐Specific Expansion of Proteins Exported to Erythrocytes in Malaria Parasites,” Genome Biology 7, no. 2 (2006): R12. 10.1186/gb-2006-7-2-r12 PMC1431722 16507167

[56] A. G. Maier , M. Rug , M. T. O'Neill , et al., “Exported Proteins Required for Virulence and Rigidity of *Plasmodium falciparum*‐Infected Human Erythrocytes,” Cell 134, no. 1 (2008): 48–61. 10.1016/j.cell.2008.04.051 PMC2568870 18614010

[57] M. Marti , R. T. Good , M. Rug , E. Knuepfer , and A. F. Cowman , “Targeting Malaria Virulence and Remodeling Proteins to the Host Erythrocyte,” Science 306, no. 5703 (2004): 1930–1933. 10.1126/science.1102452 15591202

[58] N. L. Hiller , S. Bhattacharjee , C. van Ooij , et al., “A Host‐Targeting Signal in Virulence Proteins Reveals a Secretome in Malarial Infection,” Science 306, no. 5703 (2004): 1934–1937. 10.1126/science.1102737 15591203

[59] D. S. Marapana , L. F. Dagley , J. J. Sandow , et al., “Plasmepsin V Cleaves Malaria Effector Proteins in a Distinct Endoplasmic Reticulum Translocation Interactome for Export to the Erythrocyte,” Nature Microbiology 3, no. 9 (2018): 1010–1022. 10.1038/s41564-018-0219-2 30127496

[60] A. J. Polino , M. M. Hasan , K. Floyd , Y. Avila‐Cruz , Y. Yang , and D. E. Goldberg , “An Essential Endoplasmic Reticulum‐Resident N‐Acetyltransferase Ortholog in *Plasmodium falciparum* ,” Journal of Cell Science 136, no. 6 (2023): jcs260551. 10.1242/jcs.260551 PMC10038149 36744402

[61] P. R. Gilson , S. A. Chisholm , B. S. Crabb , and T. F. de Koning‐Ward , “Host Cell Remodelling in Malaria Parasites: A New Pool of Potential Drug Targets,” International Journal for Parasitology 47, no. 2–3 (2017): 119–127. 10.1016/j.ijpara.2016.06.001 27368610

[62] J. M. Przyborski , B. Nyboer , and M. Lanzer , “Ticket to Ride: Export of Proteins to the *Plasmodium falciparum*‐Infected Erythrocyte,” Molecular Microbiology 101, no. 1 (2016): 1–11. 10.1111/mmi.13380 26996123

[63] T. Spielmann and T. W. Gilberger , “Critical Steps in Protein Export of *Plasmodium falciparum* Blood Stages,” Trends In Parasitology 31, no. 10 (2015): 514–525. 10.1016/j.pt.2015.06.010 26433254

[64] T. F. de Koning‐Ward , P. R. Gilson , J. A. Boddey , et al., “A Newly Discovered Protein Export Machine in Malaria Parasites,” Nature 459, no. 7249 (2009): 945–949. 10.1038/nature08104 PMC2725363 19536257

[65] B. Elsworth , K. Matthews , C. Q. Nie , et al., “PTEX Is an Essential Nexus for Protein Export in Malaria Parasites,” Nature 511, no. 7511 (2014): 587–591. 10.1038/nature13555 25043043

[66] J. R. Beck , V. Muralidharan , A. Oksman , and D. E. Goldberg , “PTEX Component HSP101 Mediates Export of Diverse Malaria Effectors Into Host Erythrocytes,” Nature 511, no. 7511 (2014): 592–595. 10.1038/nature13574 PMC4130291 25043010

[67] J. M. Matz , K. Matuschewski , and T. W. A. Kooij , “Two Putative Protein Export Regulators Promote *Plasmodium* Blood Stage Development In Vivo,” Molecular and Biochemical Parasitology 191, no. 1 (2013): 44–52. 10.1016/j.molbiopara.2013.09.003 24076174

[68] J. A. Boddey , M. T. O'Neill , S. Lopaticki , et al., “Export of Malaria Proteins Requires Co‐Translational Processing of the PEXEL Motif Independent of Phosphatidylinositol‐3‐Phosphate Binding,” Nature Communications 7 (2016): 10470. 10.1038/ncomms10470 PMC4740378 26832821

[69] H. H. Chang , A. M. Falick , P. M. Carlton , J. W. Sedat , J. L. DeRisi , and M. A. Marletta , “N‐Terminal Processing of Proteins Exported by Malaria Parasites,” Molecular and Biochemical Parasitology 160, no. 2 (2008): 107–115. 10.1016/j.molbiopara.2008.04.011 PMC2922945 18534695

[70] M. Rug , M. Cyrklaff , A. Mikkonen , et al., “Export of Virulence Proteins by Malaria‐Infected Erythrocytes Involves Remodeling of Host Actin Cytoskeleton,” Blood 124, no. 23 (2014): 3459–3468. 10.1182/blood-2014-06-583054 PMC4246041 25139348

[71] S. Das , “An Overview of the Interconnectedness of the PEXEL and Non‐PEXEL Mediated Protein Export in *Plasmodium falciparum* ,” iScience 28, no. 10 (2025): 113427. 10.1016/j.isci.2025.113427 PMC12483598 41035680

[72] N. Boonyalai , C. R. Collins , F. Hackett , C. Withers‐Martinez , and M. J. Blackman , “Essentiality of *Plasmodium falciparum* Plasmepsin V,” PLoS One 13, no. 12 (2018): e0207621. 10.1371/journal.pone.0207621 PMC6281190 30517136

[73] L. Gambini , L. Rizzi , A. Pedretti , et al., “Picomolar Inhibition of Plasmepsin V, an Essential Malaria Protease, Achieved Exploiting the Prime Region,” PLoS One 10, no. 11 (2015): e0142509. 10.1371/journal.pone.0142509 PMC4643876 26566224

[74] M. Gazdik , K. E. Jarman , M. T. O'Neill , et al., “Exploration of the P3 Region of PEXEL Peptidomimetics Leads to a Potent Inhibitor of the *Plasmodium* Protease, Plasmepsin V,” Bioorganic & Medicinal Chemistry 24, no. 9 (2016): 1993–2010. 10.1016/j.bmc.2016.03.027 27021426

[75] M. Gazdik , M. T. O'Neill , S. Lopaticki , et al., “The Effect of N‐Methylation on Transition State Mimetic Inhibitors of the *Plasmodium* Protease, Plasmepsin V,” MedChemComm 6, no. 3 (2015): 437–443.

[76] A. N. Hodder , B. E. Sleebs , P. E. Czabotar , et al., “Structural Basis for Plasmepsin V Inhibition That Blocks Export of Malaria Proteins to Human Erythrocytes,” Nature Structural & Molecular Biology 22, no. 8 (2015): 590–596. 10.1038/nsmb.3061 26214367

[77] W. Nguyen , A. N. Hodder , R. B. de Lezongard , et al., “Enhanced Antimalarial Activity of Plasmepsin V Inhibitors by Modification of the P2 Position of PEXEL Peptidomimetics,” European Journal of Medicinal Chemistry 154 (2018): 182–198. 10.1016/j.ejmech.2018.05.022 29800827

[78] B. E. Sleebs , M. Gazdik , M. T. O'Neill , et al., “Transition State Mimetics of the *Plasmodium* Export Element Are Potent Inhibitors of Plasmepsin V From *P. falciparum* and *P. vivax* ,” Journal of Medicinal Chemistry 57, no. 18 (2014): 7644–7662. 10.1021/jm500797g 25167370

[79] B. E. Sleebs , S. Lopaticki , D. S. Marapana , et al., “Inhibition of Plasmepsin V Activity Demonstrates Its Essential Role in Protein Export, PfEMP1 Display, and Survival of Malaria Parasites,” PLoS Biology 12, no. 7 (2014): e1001897. 10.1371/journal.pbio.1001897 PMC4077696 24983235

[80] A. J. Polino , A. S. Nasamu , J. C. Niles , and D. E. Goldberg , “Assessment of Biological Role and Insight Into Druggability of the *Plasmodium falciparum* Protease Plasmepsin V,” ACS Infectious Diseases 6, no. 4 (2020): 738–746. 10.1021/acsinfecdis.9b00460 PMC7155168 32069391

[81] C. Jennison , L. Lucantoni , M. T. O'Neill , et al., “Inhibition of Plasmepsin V Activity Blocks *Plasmodium falciparum* Gametocytogenesis and Transmission to Mosquitoes,” Cell Reports 29, no. 12 (2019): 3796–3806. 10.1016/j.celrep.2019.11.073 31851913

[82] K. Matthews , M. Kalanon , S. A. Chisholm , et al., “The *Plasmodium* Translocon of Exported Proteins (PTEX) Component Thioredoxin‐2 Is Important for Maintaining Normal Blood‐Stage Growth,” Molecular Microbiology 89, no. 6 (2013): 1167–1186. 10.1111/mmi.12334 23869529

[83] F. Silvestrini , E. Lasonder , A. Olivieri , et al., “Protein Export Marks the Early Phase of Gametocytogenesis of the Human Malaria Parasite *Plasmodium falciparum* ,” Molecular & Cellular Proteomics 9, no. 7 (2010): 1437–1448. 10.1074/mcp.M900479-MCP200 PMC2938084 20332084

[84] M. Tibúrcio , M. W. A. Dixon , O. Looker , S. Y. Younis , L. Tilley , and P. Alano , “Specific Expression and Export of the *Plasmodium falciparum* Gametocyte Exported Protein‐5 Marks the Gametocyte Ring Stage,” Malaria Journal 14 (2015): 334. 10.1186/s12936-015-0853-6 PMC4552133 26315106

[85] P. Ngotho , K. D. Press , M. Peedell , et al., “Reversible Host Cell Surface Remodelling Limits Immune Recognition and Maximizes Transmission of Plasmodium falciparum Gametocytes,” bioRxiv (2024). 10.1371/journal.ppat.1013110 PMC12091884 40354414

[86] B. J. Morahan , C. Strobel , U. Hasan , et al., “Functional Analysis of the Exported Type IV HSP40 Protein PfGECO in *Plasmodium falciparum* Gametocytes,” Eukaryotic Cell 10, no. 11 (2011): 1492–1503. 10.1128/EC.05155-11 PMC3209067 21965515

[87] S. Eksi , Y. Haile , T. Furuya , L. Ma , X. Su , and K. C. Williamson , “Identification of a Subtelomeric Gene Family Expressed During the Asexual‐Sexual Stage Transition in *Plasmodium falciparum* ,” Molecular and Biochemical Parasitology 143, no. 1 (2005): 90–99. 10.1016/j.molbiopara.2005.05.010 15996767

[88] A. M. Vaughan , S. A. Mikolajczak , E. M. Wilson , et al., “Complete *Plasmodium falciparum* Liver‐Stage Development in Liver‐Chimeric Mice,” Journal of Clinical Investigation 122, no. 10 (2012): 3618–3628. 10.1172/JCI62684 PMC3461911 22996664

[89] M. Kalanon , D. Bargieri , A. Sturm , et al., “The *Plasmodium* Translocon of Exported Proteins Component EXP2 Is Critical for Establishing a Patent Malaria Infection in Mice,” Cellular Microbiology 18, no. 3 (2016): 399–412. 10.1111/cmi.12520 26347246

[90] R. McConville , J. M. M. Krol , R. W. J. Steel , et al., “Flp/FRT‐Mediated Disruption of ptex150 and exp2 in *Plasmodium falciparum* Sporozoites Inhibits Liver‐Stage Development,” Proceedings of the National Academy of Sciences 121, no. 28 (2024): e2403442121. 10.1073/pnas.2403442121 PMC11252984 38968107

[91] G. Zanghí , H. Patel , J. L. Smith , et al., “Genome‐Wide Gene Expression Profiles Throughout Human Malaria Parasite Liver Stage Development in Humanized Mice,” Nature Microbiology 10, no. 2 (2025): 569–584. 10.1038/s41564-024-01905-5 PMC11790487 39891010

[92] D. K. Jaijyan , H. Singh , and A. P. Singh , “A Sporozoite‐ and Liver Stage‐Expressed Tryptophan‐Rich Protein Plays an Auxiliary Role in *Plasmodium* Liver Stage Development and Is a Potential Vaccine Candidate,” Journal of Biological Chemistry 290, no. 32 (2015): 19496–19511. 10.1074/jbc.M114.588129 PMC4528115 25960542

[93] Y. Orito , T. Ishino , S. Iwanaga , et al., “Liver‐Specific Protein 2: A *Plasmodium* Protein Exported to the Hepatocyte Cytoplasm and Required for Merozoite Formation,” Molecular Microbiology 87, no. 1 (2013): 66–79. 10.1111/mmi.12083 23216750

[94] F. U. Hügel , G. Pradel , and U. Frevert , “Release of Malaria Circumsporozoite Protein Into the Host Cell Cytoplasm and Interaction With Ribosomes,” Molecular and Biochemical Parasitology 81, no. 2 (1996): 151–170. 10.1016/0166-6851(96)02701-6 8898331

[95] R. McConville , R. W. J. Steel , M. T. O'Neill , A. F. Cowman , N. Kneteman , and J. A. Boddey , “The Vaccine Candidate Liver Stage Antigen 3 Is Exported During *Plasmodium falciparum* Infection and Required for Liver‐Stage Development,” eLife 14 (2025): RP108524.

[96] A. N. Hodder , B. E. Sleebs , G. Adams , et al., “Structure‐Activity Analysis of Imino‐Pyrimidinone‐Fused Pyrrolidines Aids the Development of Dual Plasmepsin V and Plasmepsin X Inhibitors,” FEBS Journal 292, no. 11 (2025): 2843–2864. 10.1111/febs.70038 PMC12138161 40035447

[97] L. Aravind , V. Anantharaman , S. Balaji , M. Babu , and L. Iyer , “The Many Faces of the Helix‐Turn‐Helix Domain: Transcription Regulation and Beyond,” FEMS Microbiology Reviews 29, no. 2 (2005): 231–262. 10.1016/j.femsre.2004.12.008 15808743

[98] S. B. P. Athauda , K. Matsumoto , S. Rajapakshe , et al., “Enzymic and Structural Characterization of Nepenthesin, a Unique Member of a Novel Subfamily of Aspartic Proteinases,” Biochemical Journal 381, no. Pt 1 (2004): 295–306. 10.1042/BJ20031575 PMC1133788 15035659

[99] A. J. Polino , J. J. Miller , S. Bhakat , et al., “The Nepenthesin Insert in the *Plasmodium falciparum* Aspartic Protease Plasmepsin V Is Necessary for Enzyme Function,” Journal of Biological Chemistry 298, no. 9 (2022): 102355. 10.1016/j.jbc.2022.102355 PMC9478907 35952758

[100] A. Ecker , E. S. C. Bushell , R. Tewari , and R. E. Sinden , “Reverse Genetics Screen Identifies Six Proteins Important for Malaria Development in the Mosquito,” Molecular Microbiology 70, no. 1 (2008): 209–220. 10.1111/j.1365-2958.2008.06407.x PMC2658712 18761621

[101] K. Müller , O. Silvie , H.‐J. Mollenkopf , and K. Matuschewski , “Pleiotropic Roles for the *Plasmodium berghei* RNA Binding Protein UIS12 in Transmission and Oocyst Maturation,” Frontiers in Cellular and Infection Microbiology 11 (2021): 624945. 10.3389/fcimb.2021.624945 PMC7973279 33747980

[102] D. G. Paton , L. M. Childs , M. A. Itoe , I. E. Holmdahl , C. O. Buckee , and F. Catteruccia , “Exposing *Anopheles* Mosquitoes to Antimalarials Blocks *Plasmodium* Parasite Transmission,” Nature 567, no. 7747 (2019): 239–243. 10.1038/s41586-019-0973-1 PMC6438179 30814727

[103] D. G. Paton , A. S. Probst , E. Ma , et al., “Using an Antimalarial in Mosquitoes Overcomes *Anopheles* and *Plasmodium* Resistance to Malaria Control Strategies,” PLoS Pathogens 18, no. 6 (2022): e1010609. 10.1371/journal.ppat.1010609 PMC9223321 35687594

[104] A. S. Probst , D. G. Paton , F. Appetecchia , et al., “In Vivo Screen of *Plasmodium* Targets for Mosquito‐Based Malaria Control,” Nature 643, no. 8072 (2025): 785–793. 10.1038/s41586-025-09039-2 PMC12267055 40399670

[105] M. L. Wilde , S. N. Farrell , C. D. Goodman , and G. I. McFadden , “Novel Techniques for Disrupting Malaria Transmission,” Trends In Parasitology 41, no. 8 (2025): 657–669. 10.1016/j.pt.2025.06.005 40645886

[106] B. S. Mastan , A. Kumari , D. Gupta , S. Mishra , and K. A. Kumar , “Gene Disruption Reveals a Dispensable Role for Plasmepsin VII in the *Plasmodium berghei* Life Cycle,” Molecular and Biochemical Parasitology 195, no. 1 (2014): 10–13. 10.1016/j.molbiopara.2014.05.004 24893340

[107] B. S. Mastan , S. K. Narwal , S. Dey , K. A. Kumar , and S. Mishra , “ *Plasmodium berghei* Plasmepsin VIII Is Essential for Sporozoite Gliding Motility,” International Journal for Parasitology 47, no. 5 (2017): 239–245. 10.1016/j.ijpara.2016.11.009 28192122

[108] P. Favuzza , M. de Lera Ruiz , J. K. Thompson , et al., “Dual Plasmepsin‐Targeting Antimalarial Agents Disrupt Multiple Stages of the Malaria Parasite Life Cycle,” Cell Host & Microbe 27, no. 4 (2020): 642–658.e12. 10.1016/j.chom.2020.02.005 PMC7146544 32109369

[109] R. W. J. Steel , Y. C. Chua , S. Caiazzo , et al., “Chemovaccination With a Novel Antimalarial Targeting the Late Liver Stage Induces Durable Immunity Against Malaria,” bioRxiv (2025): 2025.07.22.666032.

[110] G. E. Weiss , P. R. Gilson , T. Taechalertpaisarn , et al., “Revealing the Sequence and Resulting Cellular Morphology of Receptor‐Ligand Interactions During *Plasmodium falciparum* Invasion of Erythrocytes,” PLoS Pathogens 11, no. 2 (2015): e1004670. 10.1371/journal.ppat.1004670 PMC4344246 25723550

[111] E. Deu , “Proteases as Antimalarial Targets: Strategies for Genetic, Chemical, and Therapeutic Validation,” FEBS Journal 284, no. 16 (2017): 2604–2628. 10.1111/febs.14130 PMC5575534 28599096

[112] D. L. Baldi , R. Good , M. T. Duraisingh , B. S. Crabb , and A. F. Cowman , “Identification and Disruption of the Gene Encoding the Third Member of the Low‐Molecular‐Mass Rhoptry Complex in *Plasmodium falciparum* ,” Infection and Immunity 70, no. 9 (2002): 5236–5245. 10.1128/IAI.70.9.5236-5245.2002 PMC128283 12183575

[113] A. N. Hodder , J. Christensen , S. Scally , et al., “Basis for Drug Selectivity of Plasmepsin IX and X Inhibition in *Plasmodium falciparum* and *vivax* ,” Structure 30, no. 7 (2022): 947–961. 10.1016/j.str.2022.03.018 35460613

[114] L. M. Low , Y. Azasi , E. S. Sherling , et al., “Deletion of *Plasmodium falciparum* Protein RON3 Affects the Functional Translocation of Exported Proteins and Glucose Uptake,” mBio 10, no. 4 (2019): e01460‐19. 10.1128/mBio.01460-19 PMC6747712 31289187

[115] D. Ito , Y. Kondo , E. Takashima , et al., “Roles of the RON3 C‐Terminal Fragment in Erythrocyte Invasion and Blood‐Stage Parasite Proliferation in *Plasmodium falciparum* ,” Frontiers in Cellular and Infection Microbiology 13 (2023): 1197126. 10.3389/fcimb.2023.1197126 PMC10340547 37457963

[116] D. Y. Bargieri , S. Thiberge , C. L. Tay , et al., “ *Plasmodium* Merozoite TRAP Family Protein Is Essential for Vacuole Membrane Disruption and Gamete Egress From Erythrocytes,” Cell Host & Microbe 20, no. 5 (2016): 618–630. 10.1016/j.chom.2016.10.015 PMC5104695 27832590

[117] L. W. Richardson , T. D. Ashton , M. G. Dans , et al., “Substrate Peptidomimetic Inhibitors of *P. falciparum* Plasmepsin X With Potent Antimalarial Activity,” ChemMedChem 17, no. 18 (2022): e202200306. 10.1002/cmdc.202200306 PMC9804387 35906744

[118] S. Mukherjee , S. Nguyen , E. Sharma , and D. E. Goldberg , “Maturation and Substrate Processing Topography of the *Plasmodium falciparum* Invasion/Egress Protease Plasmepsin X,” Nature Communications 13, no. 1 (2022): 4537. 10.1038/s41467-022-32271-7 PMC9352755 35927261

[119] A. S. Paul and M. T. Duraisingh , “Targeting *Plasmodium* Proteases to Block Malaria Parasite Escape and Entry,” Trends in Parasitology 34, no. 2 (2018): 95–97. 10.1016/j.pt.2017.11.012 29269028

[120] M. J. Blackman , H. Fujioka , W. H. L. Stafford , et al., “A Subtilisin‐Like Protein in Secretory Organelles of *Plasmodium falciparum* Merozoites,” Journal of Biological Chemistry 273, no. 36 (1998): 23398–23409. 10.1074/jbc.273.36.23398 9722575

[121] S. Yeoh , R. A. O'Donnell , K. Koussis , et al., “Subcellular Discharge of a Serine Protease Mediates Release of Invasive Malaria Parasites From Host Erythrocytes,” Cell 131, no. 6 (2007): 1072–1083. 10.1016/j.cell.2007.10.049 18083098

[122] S. Das , N. Hertrich , A. J. Perrin , et al., “Processing of *Plasmodium falciparum* Merozoite Surface Protein MSP1 Activates a Spectrin‐Binding Function Enabling Parasite Egress From RBCs,” Cell Host & Microbe 18, no. 4 (2015): 433–444. 10.1016/j.chom.2015.09.007 PMC4608996 26468747

[123] A. Yap , M. F. Azevedo , P. R. Gilson , et al., “Conditional Expression of Apical Membrane Antigen 1 in *Plasmodium falciparum* Shows It Is Required for Erythrocyte Invasion by Merozoites,” Cellular Microbiology 16, no. 5 (2014): 642–656. 10.1111/cmi.12287 PMC4231980 24571085

[124] T. Triglia , S. W. Scally , B. A. Seager , M. Pasternak , L. F. Dagley , and A. F. Cowman , “Plasmepsin X Activates the PCRCR Complex of *Plasmodium falciparum* by Processing PfRh5 for Erythrocyte Invasion,” Nature Communications 14, no. 1 (2023): 2219. 10.1038/s41467-023-37890-2 PMC10113190 37072430

[125] P. Kesari , A. Deshmukh , N. Pahelkar , et al., “Structures of Plasmepsin X From *Plasmodium falciparum* Reveal a Novel Inactivation Mechanism of the Zymogen and Molecular Basis for Binding of Inhibitors in Mature Enzyme,” Protein Science 31, no. 4 (2022): 882–899. 10.1002/pro.4279 PMC8927862 35048450

[126] P. Favuzza , J. Palandri , M. de Lera Ruiz , et al., “MK‐7602: A Potent Multi‐Stage Dual‐Targeting Antimalarial,” EBioMedicine 123 (2026): 106061. 10.1016/j.ebiom.2025.106061 PMC12721278 41353980

[127] T. Kariu , T. Ishino , K. Yano , Y. Chinzei , and M. Yuda , “CelTOS, A Novel Malarial Protein That Mediates Transmission to Mosquito and Vertebrate Hosts,” Molecular Microbiology 59, no. 5 (2006): 1369–1379. 10.1111/j.1365-2958.2005.05024.x 16468982

[128] O. Silvie , J. F. Franetich , S. Charrin , et al., “A Role for Apical Membrane Antigen 1 During Invasion of Hepatocytes by *Plasmodium falciparum* Sporozoites,” Journal of Biological Chemistry 279, no. 10 (2004): 9490–9496. 10.1074/jbc.M311331200 14676185

[129] L. Tawk , C. Lacroix , P. Gueirard , et al., “A Key Role for *Plasmodium* Subtilisin‐Like SUB1 Protease in Egress of Malaria Parasites From Host Hepatocytes,” Journal of Biological Chemistry 288, no. 46 (2013): 33336–33346. 10.1074/jbc.M113.513234 PMC3829181 24089525

[130] R. Al‐Kaisy , S. Bhatt , and D. A. Duchêne , “Distinct Evolutionary Regimes Across Domains of the *Plasmodium falciparum* CSP Gene,” Scientific Reports 15, no. 1 (2025): 13507. 10.1038/s41598-025-98456-4 PMC12008178 40251276

[131] O. A. C. Lamers , B. M. D. Franke‐Fayard , M. Roestenberg , and J. M. M. Krol , “The Path From Early‐ to Late‐Liver Stage Arresting Genetically Attenuated Parasites as a Malaria Vaccination Strategy,” NPJ Vaccines 10, no. 1 (2025): 218. 10.1038/s41541-025-01265-z PMC12508238 41062524

[132] A. Nuwa , K. Baker , R. Kajubi , et al., “Effectiveness of Sulfadoxine–Pyrimethamine Plus Amodiaquine and Dihydroartemisinin–Piperaquine for Seasonal Malaria Chemoprevention in Uganda: A Three‐Arm, Open‐Label, Non‐Inferiority and Superiority, Cluster‐Randomised, Controlled Trial,” Lancet Infectious Diseases 25, no. 7 (2025): 726–736. 10.1016/S1473-3099(24)00746-1 39826559

[133] L. Burgert , T. Reiker , M. Golumbeanu , J. J. Möhrle , and M. A. Penny , “Model‐Informed Target Product Profiles of Long‐Acting‐Injectables for Use as Seasonal Malaria Prevention,” PLOS Global Public Health 2, no. 3 (2022): e0000211. 10.1371/journal.pgph.0000211 PMC10021282 36962305

[134] F. Macintyre , H. Ramachandruni , J. N. Burrows , et al., “Injectable Anti‐Malarials Revisited: Discovery and Development of New Agents to Protect Against Malaria,” Malaria Journal 17, no. 1 (2018): 402. 10.1186/s12936-018-2549-1 PMC6211409 30384848

[135] J. E. Johnson , A. L. Brotherton , M. R. Rossi , M. C. Sanchez , and C. G. Beckwith , “Long‐Acting Injectable Antiretroviral Therapy for Treatment of Human Immunodeficiency Virus: A Review,” Current HIV/AIDS Reports 22, no. 1 (2025): 31. 10.1007/s11904-025-00741-4 40266468

[136] P. J. Rosenthal , “Falcipains and Other Cysteine Proteases of Malaria Parasites,” Advances in Experimental Medicine and Biology 712 (2011): 30–48. 10.1007/978-1-4419-8414-2_3 21660657

[137] R. A. O'Donnell , F. Hackett , S. A. Howell , et al., “Intramembrane Proteolysis Mediates Shedding of a Key Adhesin During Erythrocyte Invasion by the Malaria Parasite,” Journal of Cell Biology 174, no. 7 (2006): 1023–1033. 10.1083/jcb.200604136 PMC2064393 17000879

[138] D. P. Rotella , “The Discovery and Development of Boceprevir,” Expert Opinion on Drug Discovery 8, no. 11 (2013): 1439–1447. 10.1517/17460441.2013.843525 24079543

[139] G. J. Tanoury , S. Eastham , C. L. Harrison , et al., “Telaprevir: From Drug Discovery to the Manufacture of Drug Substance,” Comprehensive Accounts of Pharmaceutical Research and Development: From Discovery to Late‐Stage Process Development, Volume 1 (American Chemical Society, 2016), 1239, 281–323.

[140] D. R. Owen , C. M. N. Allerton , A. S. Anderson , et al., “An Oral SARS‐CoV‐2 M(Pro) Inhibitor Clinical Candidate for the Treatment of COVID‐19,” Science 374, no. 6575 (2021): 1586–1593. 10.1126/science.abl4784 34726479

[141] A. K. Ghosh , H. L. Osswald , and G. Prato , “Recent Progress in the Development of HIV‐1 Protease Inhibitors for the Treatment of HIV/AIDS,” Journal of Medicinal Chemistry 59, no. 11 (2016): 5172–5208. 10.1021/acs.jmedchem.5b01697 PMC5598487 26799988

[142] C. Jensen , P. Herold , and H. R. Brunner , “Aliskiren: The First Renin Inhibitor for Clinical Treatment,” Nature Reviews Drug Discovery 7, no. 5 (2008): 399–410. 10.1038/nrd2550 18340340

[143] Z. Zhu , “Iminoheterocycle as a Druggable Motif: BACE1 Inhibitors and Beyond,” Trends in Pharmacological Sciences 33, no. 5 (2012): 233–240. 10.1016/j.tips.2012.02.003 22480948

[144] R. L. Webb , N. Schiering , R. Sedrani , and J. Maibaum , “Direct Renin Inhibitors as a New Therapy for Hypertension,” Journal of Medicinal Chemistry 53, no. 21 (2010): 7490–7520. 10.1021/jm901885s 20731374

[145] J. Yuan , S. Venkatraman , Y. Zheng , B. M. McKeever , L. W. Dillard , and S. B. Singh , “Structure‐Based Design of β‐Site APP Cleaving Enzyme 1 (BACE1) Inhibitors for the Treatment of Alzheimer's Disease,” Journal of Medicinal Chemistry 56, no. 11 (2013): 4156–4180. 10.1021/jm301659n 23509904

[146] R. K. Chambers , T. A. Khan , D. B. Olsen , and B. E. Sleebs , “Synthesis of Amino Heterocycle Aspartyl Protease Inhibitors,” Organic & Biomolecular Chemistry 14, no. 22 (2016): 4970–4985. 10.1039/c5ob01842k 27143279

[147] J. Barber , P. Sikakana , C. Sadler , D. Baud , J. P. Valentin , and R. Roberts , “A Target Safety Assessment of the Potential Toxicological Risks of Targeting Plasmepsin IX/X for the Treatment of Malaria,” Toxicology Research 10, no. 2 (2021): 203–213. 10.1093/toxres/tfaa106 PMC8045587 33884171

[148] H. H. J. Gerets , A. Delaunois , A. Cardenas , et al., “Assessing the Interplay Between Off‐Target Promiscuity, Cytotoxicity, and Tolerability in Rodents to Improve the Safety Profile of Novel Anti‐Malarial Plasmepsin X Inhibitors,” Toxicological Sciences 201, no. 2 (2024): 311–320. 10.1093/toxsci/kfae086 38976649

[149] B. B. Beyer , J. V. Johnson , A. Y. Chung , et al., “Active‐Site Specificity of Digestive Aspartic Peptidases From the Four Species of *Plasmodium* That Infect Humans Using Chromogenic Combinatorial Peptide Libraries,” Biochemistry 44, no. 6 (2005): 1768–1779. 10.1021/bi047886u 15697202

[150] A. Kiso , K. Hidaka , T. Kimura , et al., “Search for Substrate‐Based Inhibitors Fitting the S2’ Space of Malarial Aspartic Protease Plasmepsin II,” Journal of Peptide Science 10, no. 11 (2004): 641–647. 10.1002/psc.609 15568678

[151] A. Nezami , T. Kimura , K. Hidaka , et al., “High‐Affinity Inhibition of a Family of *Plasmodium falciparum* Proteases by a Designed Adaptive Inhibitor,” Biochemistry 42, no. 28 (2003): 8459–8464. 10.1021/bi034131z 12859191

[152] P. A. Moura , J. B. Dame , and D. A. Fidock , “Role of *Plasmodium falciparum* Digestive Vacuole Plasmepsins in the Specificity and Antimalarial Mode of Action of Cysteine and Aspartic Protease Inhibitors,” Antimicrobial Agents and Chemotherapy 53, no. 12 (2009): 4968–4978. 10.1128/AAC.00882-09 PMC2786340 19752273

[153] T. Miura , K. Hidaka , T. Uemura , et al., “Improvement of Both Plasmepsin Inhibitory Activity and Antimalarial Activity by 2‐Aminoethylamino Substitution,” Bioorganic & Medicinal Chemistry Letters 20, no. 16 (2010): 4836–4839. 10.1016/j.bmcl.2010.06.099 20634066

[154] F. J. Gamo , L. M. Sanz , J. Vidal , et al., “Thousands of Chemical Starting Points for Antimalarial Lead Identification,” Nature 465, no. 7296 (2010): 305–310. 10.1038/nature09107 20485427

[155] C. Boss , O. Corminboeuf , C. Grisostomi , et al., “Achiral, Cheap, and Potent Inhibitors of Plasmepsins I, II, and IV,” ChemMedChem 1, no. 12 (2006): 1341–1345. 10.1002/cmdc.200600223 17091526

[156] C. L. Ciana , R. Siegrist , H. Aissaoui , et al., “Novel In Vivo Active Anti‐Malarials Based on a Hydroxy‐Ethyl‐Amine Scaffold,” Bioorganic & Medicinal Chemistry Letters 23, no. 3 (2013): 658–662. 10.1016/j.bmcl.2012.11.118 23260352

[157] L. Prade , A. F. Jones , C. Boss , et al., “X‐Ray Structure of Plasmepsin II Complexed With a Potent Achiral Inhibitor,” Journal of Biological Chemistry 280, no. 25 (2005): 23837–23843. 10.1074/jbc.M501519200 15840589

[158] V. Aureggi , V. Ehmke , J. Wieland , et al., “Potent Inhibitors of Malarial Aspartic Proteases, the Plasmepsins, by Hydroformylation of Substituted 7‐Azanorbornenes,” Chemistry—A European Journal 19, no. 1 (2013): 155–164. 10.1002/chem.201202941 23161835

[159] M. J. Meyers , M. D. Tortorella , J. Xu , et al., “Evaluation of Aminohydantoins as a Novel Class of Antimalarial Agents,” ACS Medicinal Chemistry Letters 5, no. 1 (2014): 89–93. 10.1021/ml400412x PMC4027786 24900778

[160] D. Rasina , M. Otikovs , J. Leitans , et al., “Fragment‐Based Discovery of 2‐Aminoquinazolin‐4(3H)‐Ones as Novel Class Nonpeptidomimetic Inhibitors of the Plasmepsins I, II, and IV,” Journal of Medicinal Chemistry 59, no. 1 (2016): 374–387. 10.1021/acs.jmedchem.5b01558 26670264

[161] D. Rasina , G. Stakanovs , O. V. Borysov , et al., “2‐Aminoquinazolin‐4(3H)‐One Based Plasmepsin Inhibitors With Improved Hydrophilicity and Selectivity,” Bioorganic & Medicinal Chemistry 26, no. 9 (2018): 2488–2500. 10.1016/j.bmc.2018.04.012 29636223

[162] J. A. Boddey and A. F. Cowman , “Plasmodium Nesting: Remaking the Erythrocyte From the Inside Out,” Annual Review of Microbiology 67 (2013): 243–269. 10.1146/annurev-micro-092412-155730 23808341

[163] A. G. Maier , B. M. Cooke , A. F. Cowman , and L. Tilley , “Malaria Parasite Proteins That Remodel the Host Erythrocyte,” Nature Reviews Microbiology 7, no. 5 (2009): 341–354. 10.1038/nrmicro2110 19369950

[164] W. Su , W. Nguyen , G. Siddiqui , et al., “Deconvolution of the On‐Target Activity of Plasmepsin V Peptidomimetics in *Plasmodium falciparum* Parasites,” ACS Infectious Diseases 11 (2025): 3581–3594. 10.1021/acsinfecdis.5c00742 PMC12706786 41329554

[165] M. Mandal , Z. Zhu , J. N. Cumming , et al., “Design and Validation of Bicyclic Iminopyrimidinones as Beta Amyloid Cleaving Enzyme‐1 (BACE1) Inhibitors: Conformational Constraint to Favor a Bioactive Conformation,” Journal of Medicinal Chemistry 55, no. 21 (2012): 9331–9345. 10.1021/jm301039c 22989333

[166] M. Mandal , Y. Wu , J. Misiaszek , et al., “Structure‐Based Design of an Iminoheterocyclic β‐Site Amyloid Precursor Protein Cleaving Enzyme (BACE) Inhibitor That Lowers Central Aβ in Nonhuman Primates,” Journal of Medicinal Chemistry 59, no. 7 (2016): 3231–3248. 10.1021/acs.jmedchem.5b01995 26937601

[167] M. A. Lowe , A. Cardenas , J.‐P. Valentin , et al., “Discovery and Characterization of Potent, Efficacious, Orally Available Antimalarial Plasmepsin X Inhibitors and Preclinical Safety Assessment of UCB7362,” Journal of Medicinal Chemistry 65, no. 20 (2022): 14121–14143. 10.1021/acs.jmedchem.2c01336 PMC9620073 36216349

[168] R. Zogota , L. Kinena , C. Withers‐Martinez , et al., “Peptidomimetic Plasmepsin Inhibitors With Potent Anti‐Malarial Activity and Selectivity Against Cathepsin D,” European Journal of Medicinal Chemistry 163 (2019): 344–352. 10.1016/j.ejmech.2018.11.068 PMC6336538 30529637

[169] C. Withers‐Martinez , R. George , R. Ogrodowicz , et al., “Structural Plasticity of *Plasmodium falciparum* Plasmepsin X to Accommodate Binding of Potent Macrocyclic Hydroxyethylamine Inhibitors,” Journal of Molecular Biology 437, no. 10 (2025): 169062. 10.1016/j.jmb.2025.169062 40043835

[170] V. Kovada , C. Withers‐Martinez , R. Bobrovs , et al., “Macrocyclic Peptidomimetic Plasmepsin X Inhibitors With Potent In Vitro and In Vivo Antimalarial Activity,” Journal of Medicinal Chemistry 66, no. 15 (2023): 10658–10680. 10.1021/acs.jmedchem.3c00812 PMC10424242 37505188

[171] K. Sakata , M. A. Lowe , M. Xuan , et al., “Design of Novel Series of Antimalarial PMX Inhibitors With Increased Half‐Life via Molecular Property Optimization,” ACS Medicinal Chemistry Letters 14, no. 11 (2023): 1582–1588. 10.1021/acsmedchemlett.3c00404 PMC10641918 37974949

[172] M. de Lera Ruiz , P. Favuzza , Z. Guo , et al., “The Invention of WM382, a Highly Potent PMIX/X Dual Inhibitor Toward the Treatment of Malaria,” ACS Medicinal Chemistry Letters 13, no. 11 (2022): 1745–1754. 10.1021/acsmedchemlett.2c00355 PMC9661708 36385924

[173] P. Favuzza , M. G. Dans , W. Su , et al., Dual Plasmepsin IX and X Inhibitors Are Refractory to Development of Resistance,” bioRxiv (2026), 2026.03.09.710546. 10.1371/journal.ppat.1014068 PMC13568514 42709875

[174] Symposium 148, “Aspartyl Protease Inhibitors for Plasmodium and other Apicomplexan Parasites: From Screening Hits to Clinical Proof of Biology for Malaria,” American Society of Tropical Medicine & Hygiene 2025 Annual Meeting, Toronto Canada (2025).

[175] E. Stanley Susan , P. Carstens Russ , V. Liberti Maria , et al., “First‐In‐Human Safety and Pharmacokinetics of MK‐7602, the Antimalarial Inhibitor of Plasmepsins IX/X, in Single‐ and Multiple‐Ascending‐Dose Studies,” Antimicrobial Agents and Chemotherapy 70, no. 2 (2026): e01261‐25. 10.1128/aac.01261-25 PMC12888855 41532789

[176] C. McDonald , D. Smith , M. Di Cristina , G. Kannan , Z. Dou , and V. B. Carruthers , “Toxoplasma Cathepsin Protease B and Aspartyl Protease 1 Are Dispensable for Endolysosomal Protein Digestion,” mSphere 5, no. 1 (2020): e00869‐19. 10.1128/mSphere.00869-19 PMC7021471 32051238

[177] V. Polonais , M. Shea , and D. Soldati‐Favre , “ *Toxoplasma gondii* Aspartic Protease 1 Is Not Essential in Tachyzoites,” Experimental Parasitology 128, no. 4 (2011): 454–459. 10.1016/j.exppara.2011.05.003 21616070

[178] S. K. Dogga , B. Mukherjee , D. Jacot , et al., “A Druggable Secretory Protein Maturase of *Toxoplasma* Essential for Invasion and Egress,” eLife 6 (2017): e27480. 10.7554/eLife.27480 PMC5595437 28898199

[179] B. Mukherjee , F. Tessaro , J. Vahokoski , et al., “Modeling and Resistant Alleles Explain the Selectivity of Antimalarial Compound 49c Towards Apicomplexan Aspartyl Proteases,” EMBO Journal 37, no. 7 (2018): e98047. 10.15252/embj.201798047 PMC5881632 29519896

[180] M. J. Coffey , B. E. Sleebs , A. D. Uboldi , et al., “An Aspartyl Protease Defines a Novel Pathway for Export of *Toxoplasma* Proteins Into the Host Cell,” eLife 4 (2015): e10809. 10.7554/eLife.10809 PMC4764566 26576949

[181] M. J. Coffey , L. F. Dagley , S. Seizova , et al., “Aspartyl Protease 5 Matures Dense Granule Proteins That Reside at the Host‐Parasite Interface in *Toxoplasma gondii* ,” mBio 9, no. 5 (2018): e01796‐18. 10.1128/mBio.01796-18 PMC6212819 30377279

[182] P.‐M. Hammoudi , D. Jacot , C. Mueller , et al., “Fundamental Roles of the Golgi‐Associated *Toxoplasma* Aspartyl Protease, ASP5, at the Host‐Parasite Interface,” PLoS Pathogens 11, no. 10 (2015): e1005211. 10.1371/journal.ppat.1005211 PMC4608785 26473595

[183] S. Vinayak , “Recent Advances in Genetic Manipulation of *Cryptosporidium* ,” Current Opinion in Microbiology 58 (2020): 146–152. 10.1016/j.mib.2020.09.010 PMC7746576 33161368

[184] B. A. Wallbank , E. J. Smith , J. E. D. Carrasco , et al., “ *Cryptosporidium* Aspartyl Protease 2 Is Required for Host Cell Egress of Merozoites and Male Gametes,” Molecular Biology of the Cell 36, no. 11 (2025): ar133. 10.1091/mbc.E25-06-0306 PMC12562021 40901734

[185] J. E. Dumaine , A. Sateriale , A. R. Gibson , et al., “The Enteric Pathogen *Cryptosporidium parvum* Exports Proteins Into the Cytosol of the Infected Host Cell,” eLife 10 (2021): e70451. 10.7554/eLife.70451 PMC8687662 34866573

[186] E. Rodrigues , M. A. Pallett , L. C. Straker , et al., “ *Cryptosporidium* Modifies Intestinal Microvilli Through an Exported Virulence Factor,” Cell Host & Microbe 33, no. 5 (2025): 719–730.e5. 10.1016/j.chom.2025.04.001 PMC7618952 40300595

[187] L. Chng , S. Seizova , K. Wiradiputri , et al., “Identification and Characterisation of Cryptosporidium Effector Proteins in Host‐Pathogen Interaction,” 15th Lorne Infection and Immunity Conference (2025).

[188] P. Šnebergerová , P. Bartošová‐Sojková , M. Jalovecká , and D. Sojka , “Plasmepsin‐Like Aspartyl Proteases in *Babesia* ,” Pathogens 10, no. 10 (2021): 1241. 10.3390/pathogens10101241 PMC8540915 34684190

[189] S. Gohil , L. M. Kats , T. Seemann , K. M. Fernandez , G. Siddiqui , and B. M. Cooke , “Bioinformatic Prediction of the Exportome of *Babesia bovis* and Identification of Novel Proteins in Parasite‐Infected Red Blood Cells,” International Journal for Parasitology 43, no. 5 (2013): 409–416. 10.1016/j.ijpara.2013.01.002 23395698

[190] K. G. Pellé , R. H. Y. Jiang , P. Y. Mantel , et al., “Shared Elements of Host‐Targeting Pathways Among Apicomplexan Parasites of Differing Lifestyles,” Cellular Microbiology 17, no. 11 (2015): 1618–1639. 10.1111/cmi.12460 25996544

[191] H. Hakimi , T. J. Templeton , M. Sakaguchi , et al., “Novel Babesia Bovis Exported Proteins That Modify Properties of Infected Red Blood Cells,” PLoS Pathogens 16, no. 10 (2020): e1008917. 10.1371/journal.ppat.1008917 PMC7561165 33017449

[192] B. Elsworth , C. D. Keroack , Y. Rezvani , et al., “ *Babesia divergens* Host Cell Egress Is Mediated by Essential and Druggable Kinases and Proteases,” Nature Microbiology 11, no. 2 (2026): 492–506. 10.1038/s41564-025-02238-7 PMC12872469 41593365

[193] D. Sojka , D. Hartmann , P. Bartošová‐Sojková , and J. Dvořák , “Parasite Cathepsin D‐Like Peptidases and Their Relevance as Therapeutic Targets,” Trends in Parasitology 32, no. 9 (2016): 708–723. 10.1016/j.pt.2016.05.015 27344362

